# Strategic Modulation of CO Intermediate Desorption Dynamics on Bimetallic Ni_x_Cu_y_@NC Catalyst: Synergistic Electrocatalysis for Sustainable CO_2_ Conversion

**DOI:** 10.1002/smll.202505306

**Published:** 2025-06-06

**Authors:** Jian Zhu, Guangchao Li, Anna Rokicińska, Zhenyu Wang, Piotr Kuśtrowski, Zhouguang Lu, Shoubhik Das, Pegie Cool

**Affiliations:** ^1^ Department of Chemistry University of Antwerp Antwerp 2610 Belgium; ^2^ Department of Chemistry University of Bayreuth 95447 Bayreuth Germany; ^3^ School of Metallurgy and Environment Central South University Changsha 410083 China; ^4^ Faculty of Chemistry Jagiellonian University Kraków 30‐387 Poland; ^5^ Department of Materials Science and Engineering Southern University of Science and Technology Shenzhen 518055 China

**Keywords:** bimetallic catalyst, *d*‐band center position, desorption dynamics, electrochemical CO_2_ reduction, electronic structure

## Abstract

Bimetallic catalysts are appealing for electrochemical CO_2_ reduction reaction (ECO2RR), yet the introduction of bimetallic sites leads to an incomprehensive understanding of the metal atom interaction and catalytic mechanism. In this study, a series of bimetallic Ni*_x_* Cu*_y_* @NC catalysts with varied Ni to Cu weight ratios are prepared. The as‐prepared Ni_2_Cu_1_@NC catalyst shows high carbon monoxide (CO) Faradaic efficiencies (FE_CO_) over 90% in a broad potential range of −0.7 to −1.1 V (vs reversible hydrogen electrode (RHE)) with an exceptional durability of CO selectivity over 80% and a high partial current density of −44 mA cm^−2^ at an extremely high potential of −1.3 V (vs RHE). The distinguished CO selectivity and activity are primarily attributed to the integration of Ni and Cu, which lowers the *d*‐band center position and reconstructs the electronic structure according to the valence band spectra. More specifically, the downshifted *d*‐band center position weakens the interaction strength with the *CO intermediate on the Ni_2_Cu_1_@NC catalyst's surface during the ECO2RR process, resulting in fast *CO desorption and high CO selectivity. This study provides researchers with a new insight for designing and optimizing the electrocatalysts for ECO2RR.

## Graphical abstract

A bimetallic catalyst is obtained by one‐step carbonization of in situ loaded NiCuPPc@g‐C_3_N_4_. Introducing a secondary active component can modulate the electronic structure of the primary catalyst by downshifting its *d*‐band center position, significantly lowering the CO desorption energy barrier. Thereby enabling fast CO generation dynamics and superior Faraday efficiency in electrocatalytic CO_2_ reduction.

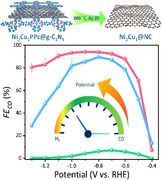

## 1 Introduction

Carbon monoxide (CO) is known as an important base chemical in industry, which can not only be directly used as a chemical feedstock but can also be further reduced into value‐added products such as methanol, ethylene, acetic, etc., at high reaction rates and high product selectivity.^[^ ^1^, ^2^ ^]^ However, the production of CO is a highly energy‐consuming process. The electrochemical CO_2_ reduction to CO presents a promising strategy not only for carbon utilization, as it can be powered by renewable energies, but also can be operated under mild conditions.^[^ ^3^, ^4^ ^]^ Whereas, the extremely stable C═O bond, multiple proton‐involved electron‐transfer steps, and competitive water splitting reaction during the reduction process usually result in sluggish kinetics and low product selectivity.^[^ ^5^, ^6^ ^]^ Considerable studies have been devoted to Pd‐,^[^ ^7^, ^8^, ^9^ ^]^ Ag‐,^[^ ^10^, ^11^, ^12^ ^]^ and Au‐based^[^ ^13^, ^14^ ^]^ electrocatalysts for CO production for their strong interactions with CO_2_ molecule and relatively weak interaction strength with the ^*^CO intermediate. However, those materials are suffering from low availability, high costs, which hinders their commercial applications.

**Figure 1:**
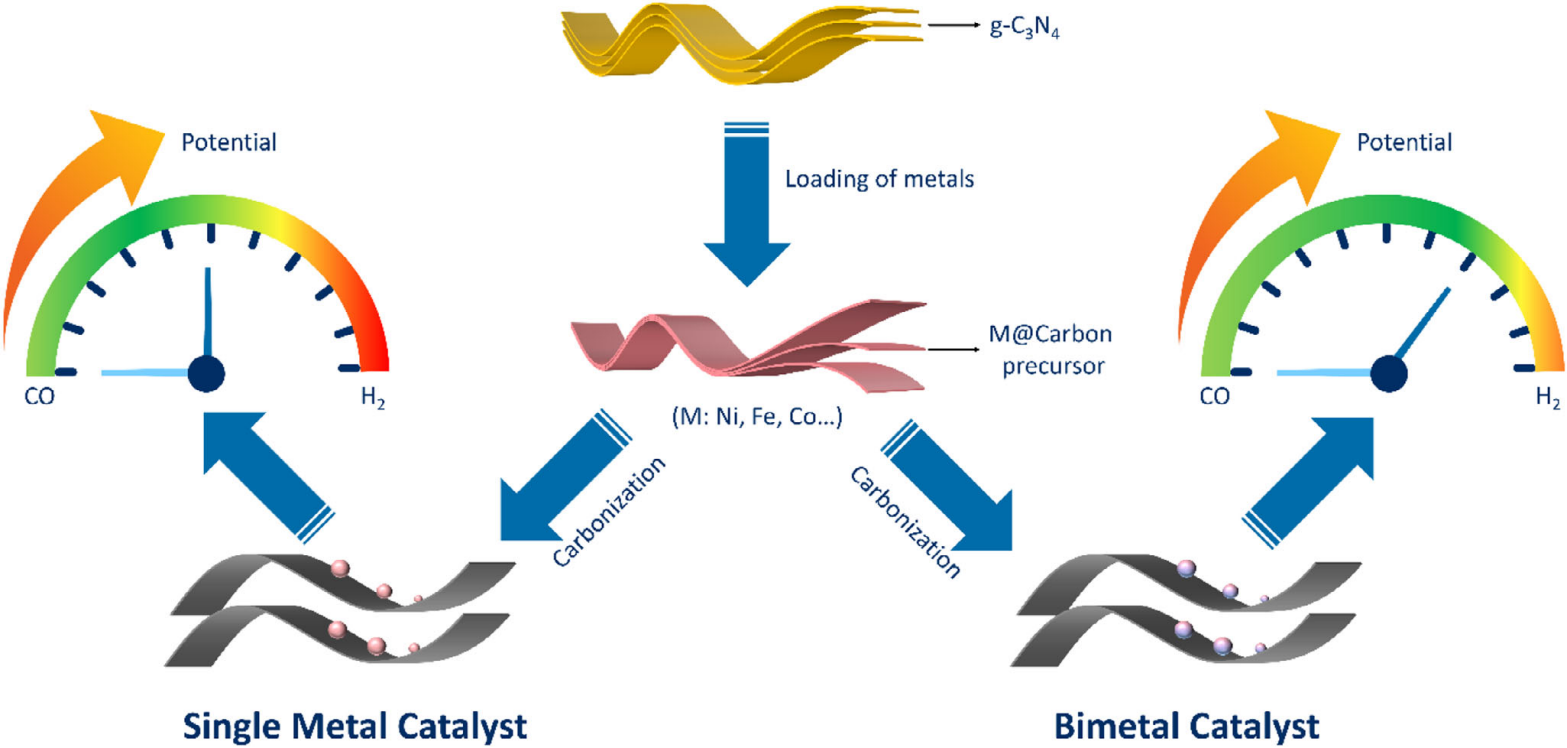
Illustration of the changes in product selectivity with the decrease of applied potential. a) monometallic catalysts, b) bimetallic catalysts.

To address these challenges, transition metal‐based electrocatalysts have been recently thoroughly investigated for ECO2RR due to their high natural abundance and tunable *3d* electronic structure. However, the ECO2RR performances of the catalysts are restricted by the linear scaling relationship of the binding energy of reaction intermediates due to the complicated multiple proton‐coupled electron transfer steps, where the binding strength with the *COOH and *CO intermediate becomes either weaker or stronger.^[^ ^15^, ^16^, ^17^ ^]^ For instance, Fe single‐atom catalysts (SAC) usually exhibit a low onset potential, whereas they suffer from detrimental hydrogen evolution reaction (HER) at more negative applied potentials due to the stronger interaction with the ^*^CO intermediate. In addition, Ni SACs often present a high overpotential, although they possess high intrinsic ECO2RR activity and relatively higher Faradaic efficiency (FE) for the CO production.^[^ ^18^ ^]^ Considerable strategies, such as controlling morphologies and heteroatom doping, have been employed to improve electrochemical performance.^[^ ^19^, ^20^, ^21^, ^22^ ^]^ However, those monometallic catalysts usually display high H_2_ selectivity when the applied potential decreases to more negative potentials, as illustrated in **Figure** **1**a (left). In contrast, incorporating a secondary metal that possesses a high intrinsic catalytic capability to form a bimetallic catalyst often results in distinct electronic structure and chemical properties, which are different from their monometallic counterparts and usually suggest a unique product selectivity.^[^ ^23^, ^24^ ^]^ This strategy offers an opportunity for designing new catalysts and optimizing binding strength with the intermediate due to the synergistic effects between the two constituent metals. This synergistic effect primarily arises from the ligand, geometric, and ensemble effects, which can contribute to high catalytic performance as demonstrated in Figure 1b.^[^ ^25^, ^26^, ^27^ ^]^ Therefore, bimetallic catalysts with tunable electronic structure are vital for optimizing binding strength, which can be realized by forming hetero‐interfaces,^[^ ^28^, ^29^ ^]^ alloys,^[^ ^30^, ^31^ ^]^ and surface modifications.^[^ ^32^ ^]^ Particularly, neither too strong nor too weak interactions with the intermediates can be achieved, which indicates that the binding energy with the intermediates can be synergistically tailored by unique electronic interactions between the two hetero‐metal species.^[^ ^33^ ^]^ For instance, by combining Cu with Au, the X‐ray photoelectron spectroscopy (XPS) spectra revealed that the *d*‐band center gradually downshifted from Cu toward Au with the increase of Au content, resulting in weakened binding of *COOH and *CO intermediates.^[^ ^26^ ^]^ In addition, by controlling the alloying metals (Ag, Cu, Zn, Sn) with Pd for the production of C1 products, the *d*‐band center position was decreased to a moderate level that facilitates the accommodation of *COOH and desorption of *CO during CO production for Sn and Ag.^[^ ^34^ ^]^ So far, various bimetallic catalysts by combining with Cu have been explored for improving selectivity and activity of ECO2RR due to the unique properties of Cu and the synergistic electronic effect. For example, Cu─M (M═Au,^[^ ^35^, ^36^, ^37^ ^]^ Pd,^[^ ^38^, ^39^ ^]^ In,^[^ ^40^ ^]^ Zn^[^ ^41^ ^]^) for the production of CO, Cu─Ag for the production of carbohydrates,^[^ ^28^, ^29^, ^32^ ^]^ Cu─Sn for the formation of formic acid,^[^ ^42^, ^43^ ^]^ and etc.^[^ ^38^, ^44^ ^]^ However, most of the reported bimetallic catalysts are blended with a noble metal and display a low current density. Although transitional bimetallic catalysts for ECO2RR were studied, severe hydrogen evolution side reactions limited the product selectivity due to the relatively stronger binding strength with ^*^CO.^[^ ^45^, ^46^ ^]^ Moreover, the practical applications are blocked by ambiguous active sites, complicated electronic structures, and preparation on a large scale. Therefore, it is desirable to develop a Ni‐based bimetallic catalyst that combines with an earth‐abundant transition metal and may deliver exceptional ECO2RR activity.

In this regard, in this study, a series of Ni*_x_* Cu*_y_* @NC bimetallic catalysts were prepared by carbonizing Ni*_x_* Cu*_y_* polyphthalocyanine @ graphitic‐carbon nitride (Ni_2_Cu_1_PPc@g‐C_3_N_4_) precursors obtained by the in situ polymerization reaction of pyromellitic dianhydride monomer on g‐C_3_N_4_ substrate. A moderate CO binding strength was demonstrated by CO temperature programmed desorption (CO‐TPD) by screening out the weight ratio of Ni to Cu, which can mitigate the poisoning of CO while maintaining high activity of ECO2RR on Ni*_x_* Cu*_y_* @NC surface. With the weight of 2:1 of Ni to Cu, the Ni_2_Cu_1_@NC catalyst displays outstanding catalytic performances for the production of CO with a high CO partial current density ((*j_CO_*)  −44 mA cm^−2^ at −1.3 V (vs reversible hydrogen electrode (RHE)) and Faradaic efficiency (>90%) in a wide potential range from −0.7 to −1.1 V (vs RHE) in a H‐cell. valence band‐XPS (VB‐XPS) verifies that incorporating Cu into Ni nanoparticles lowers the *d* band center position, which weakens the binding strength of the *CO intermediate to the Ni_2_Cu_1_@NC surface due to increased occupancy of anti‐bonding states. The fundamental understanding of the interaction behavior between Ni and Cu will shed new light on upgrading the performance of non‐noble bimetallic catalysts in ECO2RR.

## 2 Results and Discussion

### 2.1 Synthesis and Characterization of Ni*_x_* Cu*_y_* @NC Bimetallic Catalysts

Ni*_x_* Cu*_y_* @NC catalysts were prepared via a facile two‐step heat‐treatment process. Firstly, a series of Ni*_x_* Cu*_y_* PPc bimetallic precursors were prepared and in situ loaded onto g‐C_3_N_4_ substrate in a muffle furnace by a one‐step solid‐phase method with slight modifications.^[^ ^47^ ^]^ During the synthesis process, the phthalocyanine monomer was polymerized by coupling with the functional groups of pyromellitic dianhydride (PMDA). Afterward, the obtained greenish powder was washed with H_2_O and acetone several times to remove the unreacted species and residual impurities. The final catalysts with varied weight ratios of Ni to Cu were obtained by carbonizing the as‐obtained Ni_x_Cu_y_PPc@g‐C_3_N_4_ precursors under argon (Ar) atmosphere at 800 °C. As displayed in Figure S1 (Supporting Information), NiPPc@g‐C_3_N_4_, Ni_2_Cu_1_PPc@g‐C_3_N_4_, Ni_1_Cu_1_PPc@g‐C_3_N_4_, Ni_1_Cu_2_PPc@g‐C_3_N_4_, and CuPPc@g‐C_3_N_4_ precursors show the same X‐ray diffraction (XRD) patterns as those PPc‐based materials that were reported previously, manifesting the successful preparation and in situ loading of the polyphthalocyanine framework onto g‐C_3_N_4_ substrate.^[^ ^48^, ^49^, ^50^, ^51^ ^]^ In addition, Fourier transform infrared (FTIR) was applied to investigate the structure of Ni_2_Cu_1_PPc after the in situ loading of which onto the g‐C_3_N_4_ substrate. As shown in Figure S2 (Supporting Information), the absorption bands located at 806 cm^−1^ and 890 cm^−1^ in the g‐C_3_N_4_ sample correspond to the characteristic breathing mode of tri‐s‐triazine units of g‐C_3_N_4_, which is attributed to the deformation mode of N─H bonds. The other bands located between 1245 cm^−1^ and 1635 cm^−1^ in the as‐prepared samples are indexed to the typical stretching modes of C─N heterocycles.^[^ ^52^ ^]^ A broad band that is discovered between 3000 cm^−1^ and 3300 cm^−1^ is assigned to the N─H stretching vibration of the incomplete condensed amine groups and adsorbed H_2_O molecules.^[^ ^53^, ^54^, ^55^, ^56^ ^]^ The newly observed peaks positioned at 1695 cm^−1^ and 1772 cm^−1^ in the as‐prepared Ni*_x_* Cu*_y_* PPc@g‐C_3_N_4_ samples are attributed to C═N and C═C bonds, further indicating the successful in situ loading of Ni*_x_* Cu*_y_* PPc onto the g‐C_3_N_4_.^[^ ^57^, ^58^, ^59^ ^]^ Moreover, Ni*_x_* Cu*_y_* PPc samples exhibit a wide Raman band between 1514 and 1545 cm^−1^, which is attributed to the interactions between the metals and phthalocyanine ring.^[^ ^60^ ^]^ The other bands of Ni*_x_* Cu*_y_* PPc@g‐C_3_N_4_ observed in the Raman spectra are consistent with the previous results, further confirming the successful preparation of Ni*_x_* Cu*_y_* PPc@g‐C_3_N_4_ as well.^[^ ^51^, ^61^ ^]^

**Figure 2:**
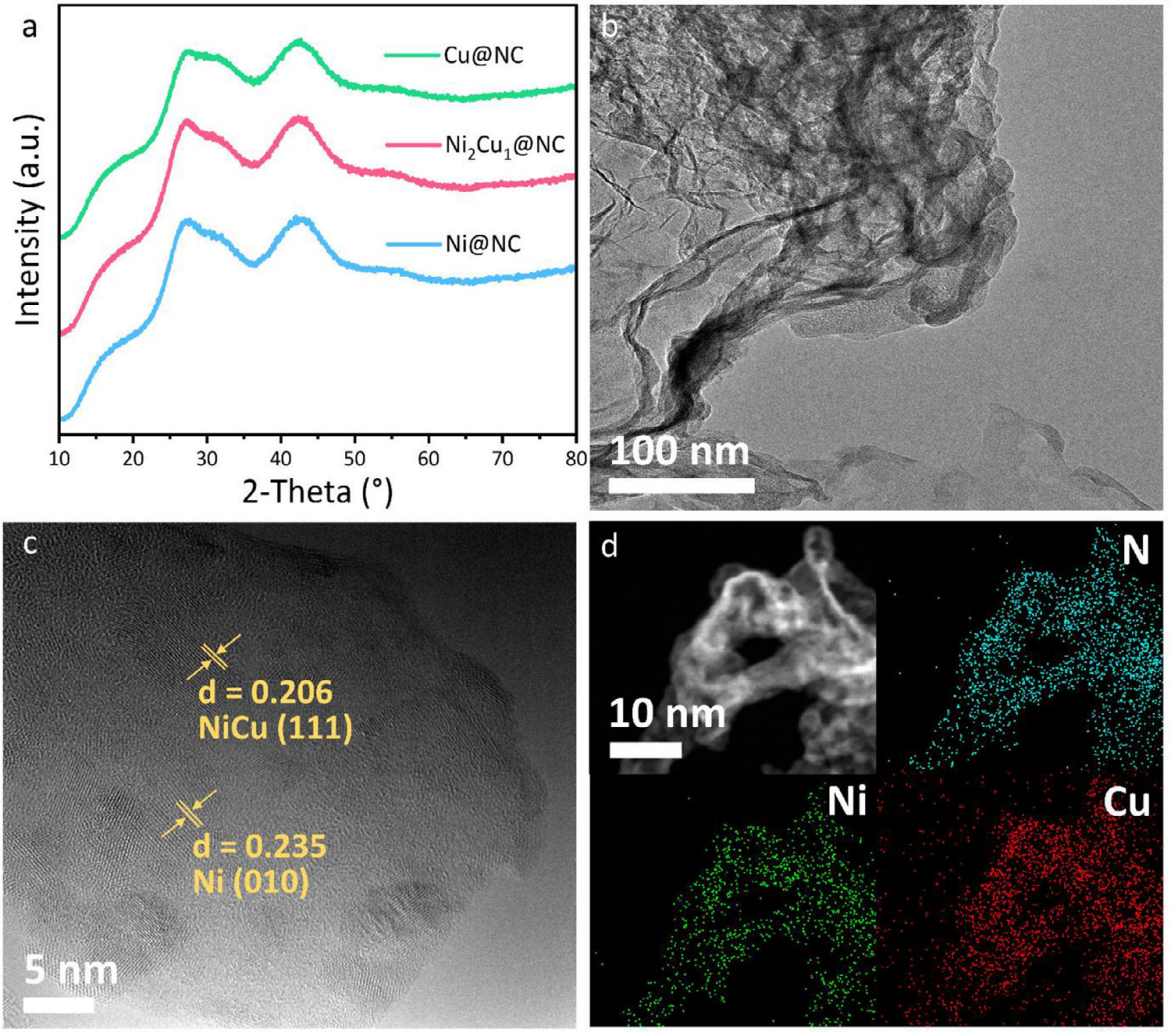
a) XRD pattern of the as‐obtained samples; b) TEM image of Ni_2_Cu_1_@NC; c) High‐resolution TEM image of Ni_2_Cu_1_@NC; d) Elemental mapping of Ni_2_Cu_1_@NC.

In order to get the final products, pyrolysis at 800 °C for 2 h under Ar was conducted. As shown in **Figures** **2**a and S4 (Supporting Information), no typical diffraction peaks corresponding to Ni‐ and Cu‐based phases are observed except two broad peaks related to carbon materials, suggesting a high dispersity of incorporated metals.^[^ ^62^ ^]^ Then, transmission electron microscopy (TEM) was employed to explore the morphologies of the as‐obtained catalysts. Figure 2b displays that the as‐prepared samples are graphene‐like layers, and no nanoparticles are observed at a low magnification, implying an ultra‐small size distribution of the supported metals. When taking a closer look at the as‐obtained samples, a lot of nanoparticles with the size distributing between 3 and 6 nm (Figure S5, Supporting Information) are observed in the Ni_2_Cu_1_@NC sample shown Figure 2c, and the lattice fringes are dominated by the (111) facets of NiCu nanoalloy with trace amount of Ni nanoparticles, respectively. The existence of Ni nanoparticles can be attributed to the relatively higher proportion of introduced Ni salt during the synthesis process of the Ni_2_Cu_1_PPc@g‐C_3_N_4_ precursor. Moreover, the selected area electron diffraction (SAED) pattern displayed in Figure S6 (Supporting Information) indicates the (200) facet of NiCu alloy, while no Ni diffraction pattern is observed. Energy‐dispersive X‐ray (EDX) images in Figure 2d identify the homogeneous distribution of N, Ni, and Cu, further indicating the ultra‐small size of NiCu nanoalloy. Figure S7 (Supporting Information) suggests similar graphene‐like morphologies for Ni@NC and Cu@NC catalysts, and the lattice fringes correspond to the (011) and (111) facets of metallic Ni and Cu, respectively. Interestingly, a much higher crystal lattice density is observed in the Cu@NC sample than in the Ni@NC sample due to the higher mobility of Cu at a high temperature. The element mapping images of the obtained samples (Figure S7c,f, Supporting Information) show the homogeneous distribution of C, N, Ni, and Cu in the graphene‐like matrix, demonstrating the successful preparation of Ni*_x_* Cu*_y_* supported on N‐doped graphene‐like carbon substrate. Besides, the inductively coupled plasma‐mass spectrometry (ICP‐MS) analysis was performed, and the proportion of the metals was determined to be ≈10%, and the weight ratio of Ni to Cu is in good agreement with the designed protocol (Table S1, Supporting Information). In addition, Raman was applied to investigate the graphitization degree of the carbon matrix. The peaks ≈1360 and 1562 cm^−1^ in Figure S8 (Supporting Information) correspond to the D band and G band. Despite the variations in metal weight ratios, the I_D_/I_G_ values show little difference between different samples, indicating similar doping environments in the carbon matrix between different catalysts. N_2_ adsorption−desorption analysis was employed to determine the specific surface area of the obtained samples. It is found in Figure S9 (Supporting Information) that Ni_2_Cu_1_@NC shows the highest specific surface area, while the other as‐obtained samples display comparable values.

**Figure 3:**
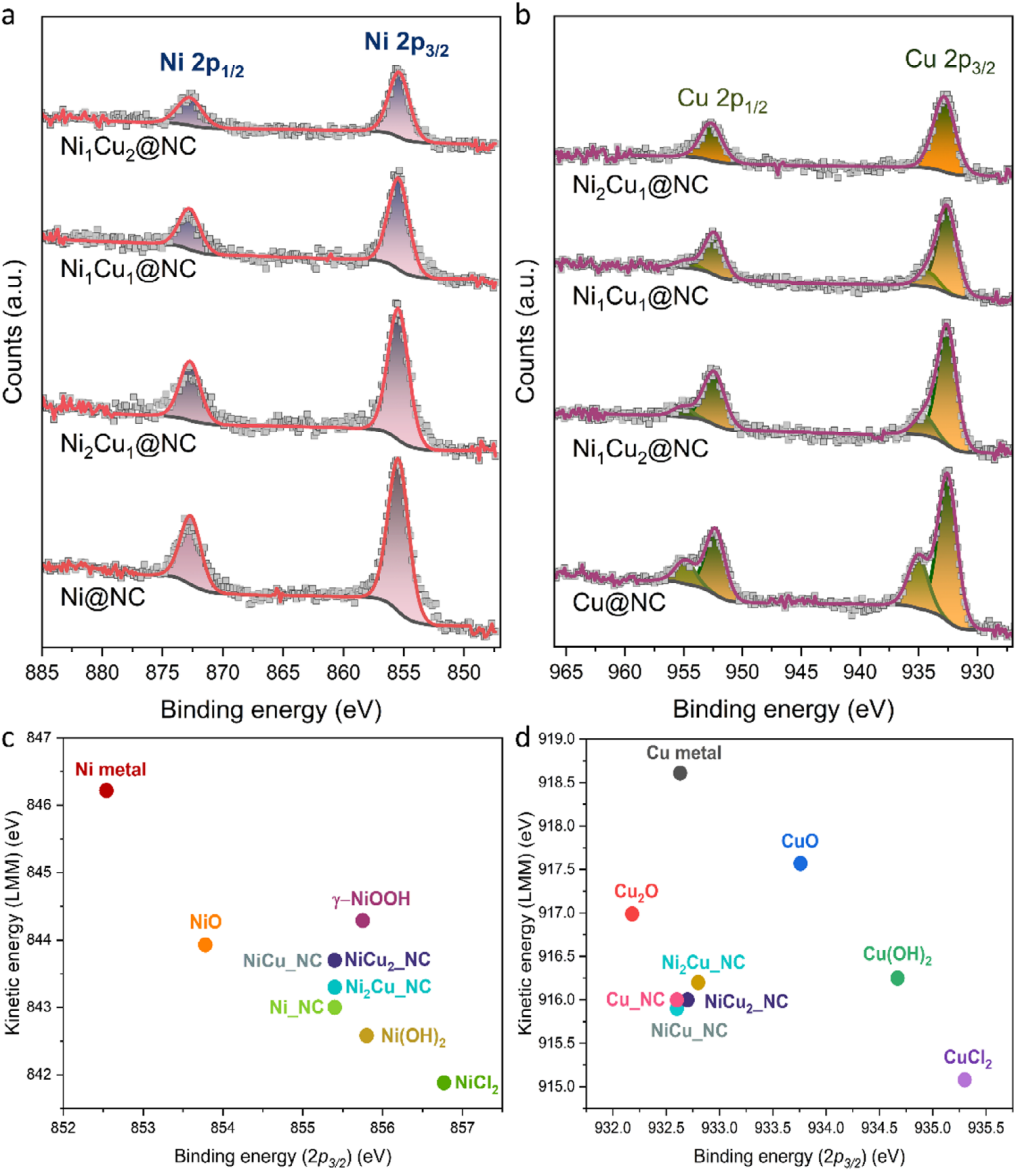
a,b) High‐resolution of Ni 2p XPS and Cu 2p XPS spectra of Ni_2_Cu_1_@NC, respectively; c) The Wagner plots of Ni LMM of the as‐prepared samples; d) The Wagner plots of Cu LMM of the as‐prepared samples.

The surface compositions of the as‐prepared samples were carefully examined by XPS measurements. As shown in Figure S10a,b (Supporting Information), the N 1s region was fitted with four peaks at 398.6, 399.8, 401.1, and 403.0 eV, corresponding to pyridinic N, pyrrolic N, graphitic N, and oxidized N, respectively.^[^ ^63^, ^64^, ^65^ ^]^ The as‐obtained samples display a similar N content of ≈21 at% (Figure S10c, Supporting Information) with little difference in total content of pyridinic N and pyrrolic N (Figure S10c,d, Supporting Information), which are usually regarded as one of the factors for promoting ECO2RR. This result suggests that the roles of N doping, which may result in differences in electrochemical performances, are negligible in this study. **Figure** **3** **a** shows that the binding energies of Ni 2p_3/2_ and Ni 2p_1/2_ peaks at 855.4 and 872.8 eV corresponding to Ni^2+^, remained regardless of the increase in Cu content, whereas the Ni content decreases with the increase in Cu content (Table S2, Supporting Information).^[^ ^66^, ^67^ ^]^ In contrast, the XPS Cu 2p spectra are deconvoluted into multiple peaks, and the binding energies shift with increasing Ni content (Table S2, Supporting Information). The peaks at 932.6 and 952.4 eV in Ni_2_Cu_1_@NC in Figure 3b are attributed to Cu 2p_3/2_ and Cu 2p_1/2_ of Cu^+^, which is additionally confirmed by the lack of characteristic shake‐up features typical for Cu^2+^.^[^ ^68^ ^]^ In addition, the content of Cu^2+^ at 934.2 and 954.9 eV resulting from the oxidation of Cu increases as the Cu content increases,^[^ ^15^, ^18^, ^69^ ^]^ The geometric area of the Cu^+^ peak is significantly greater than that of Cu^2+^, indicating that Cu_2_O is the majority component in the as‐obtained samples (Table S2, Supporting Information). To further confirm the oxidation state of the as‐prepared samples, the positions of the Auger Cu LMM and Ni LMM peaks were analyzed as well. The Wagner plots in Figure 3c,d identify the dominant Ni^2+^ and Cu^+^ species while with higher relative Ni and Cu content, respectively.

### 2.2 ECO2RR Performance of Ni*_x_* Cu*_y_* @NC Bimetallic Catalysts

**Figure 4:**
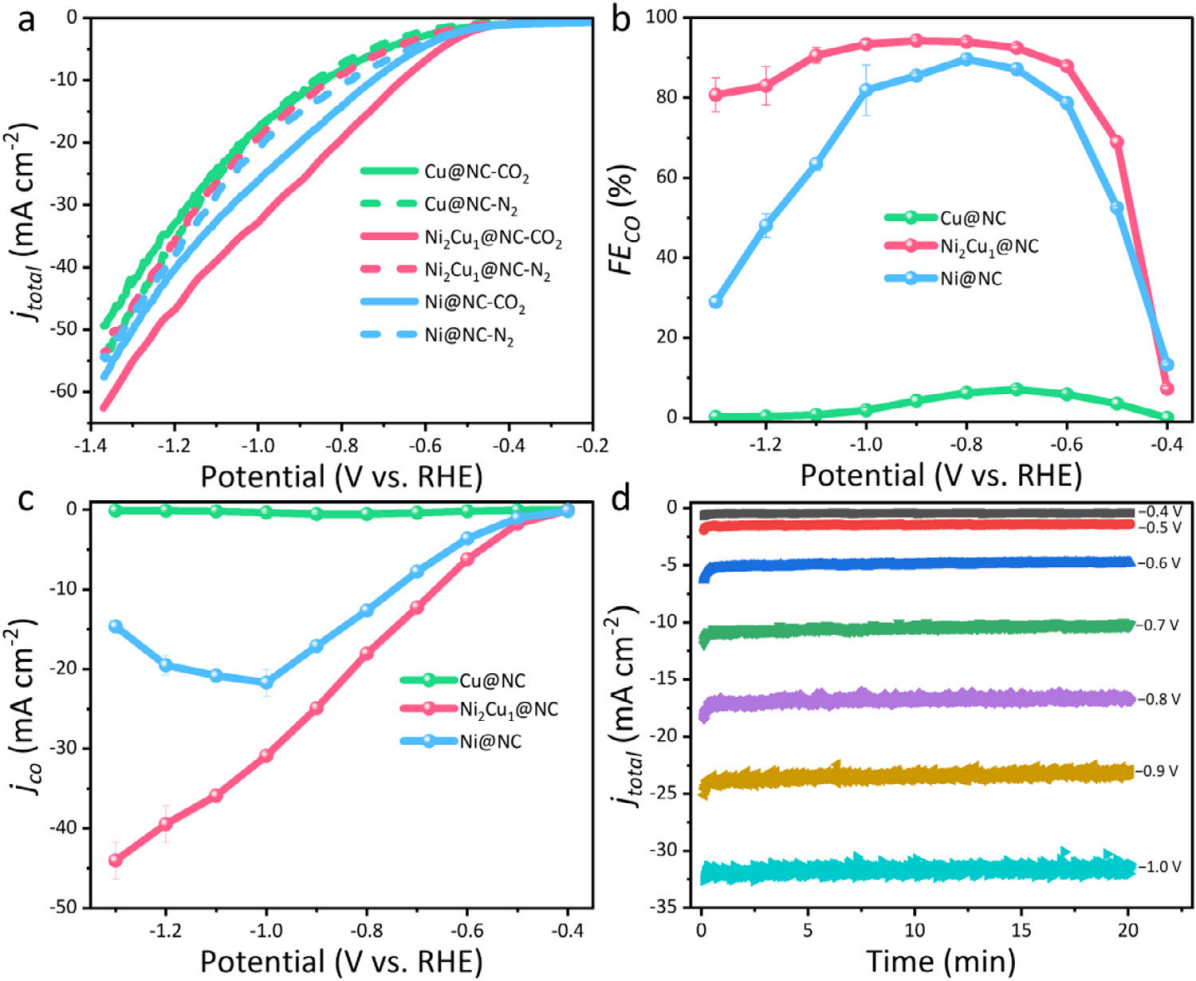
a) ECO2RR performances of the Ni*_x_* Cu*_y_* @NC catalysts. a) LSV curves in N_2_ and CO_2_ saturated 0.5 m KHCO_3_ at a scan rate of 10 mV s^−1^; b) and c) CO FEs and *j_CO_* at various potentials; d) Electrocatalysis of Ni_2_Cu_1_@NC at various potentials.

The electrocatalytic activities of these bimetallic samples were evaluated in an H‐Cell using CO_2_‐ and N_2_‐saturated 0.5 m KHCO_3_ solution as the electrolyte, and all the potentials are converted to RHE unless otherwise stated. As displayed by the linear sweep voltammetry (LSV) curves in **Figures** **4**a and S11 (Supporting Information), Ni*_x_* Cu*_y_* @NC samples exhibit higher current densities and more positive onset potentials in CO_2_‐saturated KHCO_3_ solution compared to those in N₂‐saturated KHCO₃ solution, suggesting a higher activity toward ECO2RR than HER. Particularly, the bimetallic Ni_2_Cu_1_@NC catalyst displays the highest current density in the potential range of −0.4 to −1.4 V when compared with the single metallic Ni@NC and Cu@NC catalysts, indicating the highest activity for ECO2RR. By quantifying and qualifying the liquid and gas products using H^1^ NMR and online gas chromatography (GC), CO and H_2_ were determined to be the dominant products, with no detectable liquid products (Figure S12, Supporting Information). To evaluate the competition between ECO2RR and HER, the selectivity for CO production was assessed by the FEs of CO (Figure 4b). Ni_2_Cu_1_@NC exhibits a high FE_CO_ over 90% across a wide applied potential range from −0.7 to −1.1 V, achieving a maximum FE_CO_ of 94.3% at −0.9 V. In stark contrast, much inferior ECO2RR performances are demonstrated in the Ni@NC catalyst with FE_CO_ decreasing rapidly after reaching the highest FE_CO_ of 89.6% at −0.8 V. Moreover, Cu@NC displays little promise for ECO2RR. The outstanding electrochemical performance for Ni_2_Cu_1_@NC is primarily attributed to the incorporation of Ni and Cu, which increases the CO selectivity and suppresses the HER during the ECO2RR process. In addition, the same trend as Ni@NC is observed for Ni_1_Cu_1_@NC and Ni_1_Cu_2_@NC, although the highest FE_CO_ of 89.5% for Ni_1_Cu_2_@NC is achieved at −0.8 V, as shown in Figure S13 (Supporting Information). The superiority in ECO2RR performances on the Ni_2_Cu_1_@NC catalyst could also be identified by the CO *j_CO_* as demonstrated in Figure 4c. Specifically, the bimetallic Ni_2_Cu_1_@NC catalyst delivers a much higher *j_CO_* of −24.9 mA cm^−2^ at −0.9 V and −44 mA cm^−2^ at −1.3 V than its counterparts including both monometallic and bimetallic catalysts as displayed in Figure S14 (Supporting Information), demonstrating its outstanding reactivity and selectivity for CO production which superior to the catalysts that are previously reported in the literature (Table S3, Supporting Information). In addition, the as‐obtained catalysts display stable current density at various applied potentials for 20 min as displayed in Figure 4d, whereas the FE_CO_ decreases quickly to 70% when the long‐term electrolysis proceeds as shown in Figure S15 (Supporting Information), which is attributed to the stacking of the graphene‐like layers due to the strong van der Waals forces.^[^ ^70^, ^71^ ^]^

### 2.3 ECO2RR Mechanism on Ni_2_Cu_1_@NC Bimetallic Catalysts

**Figure 5:**
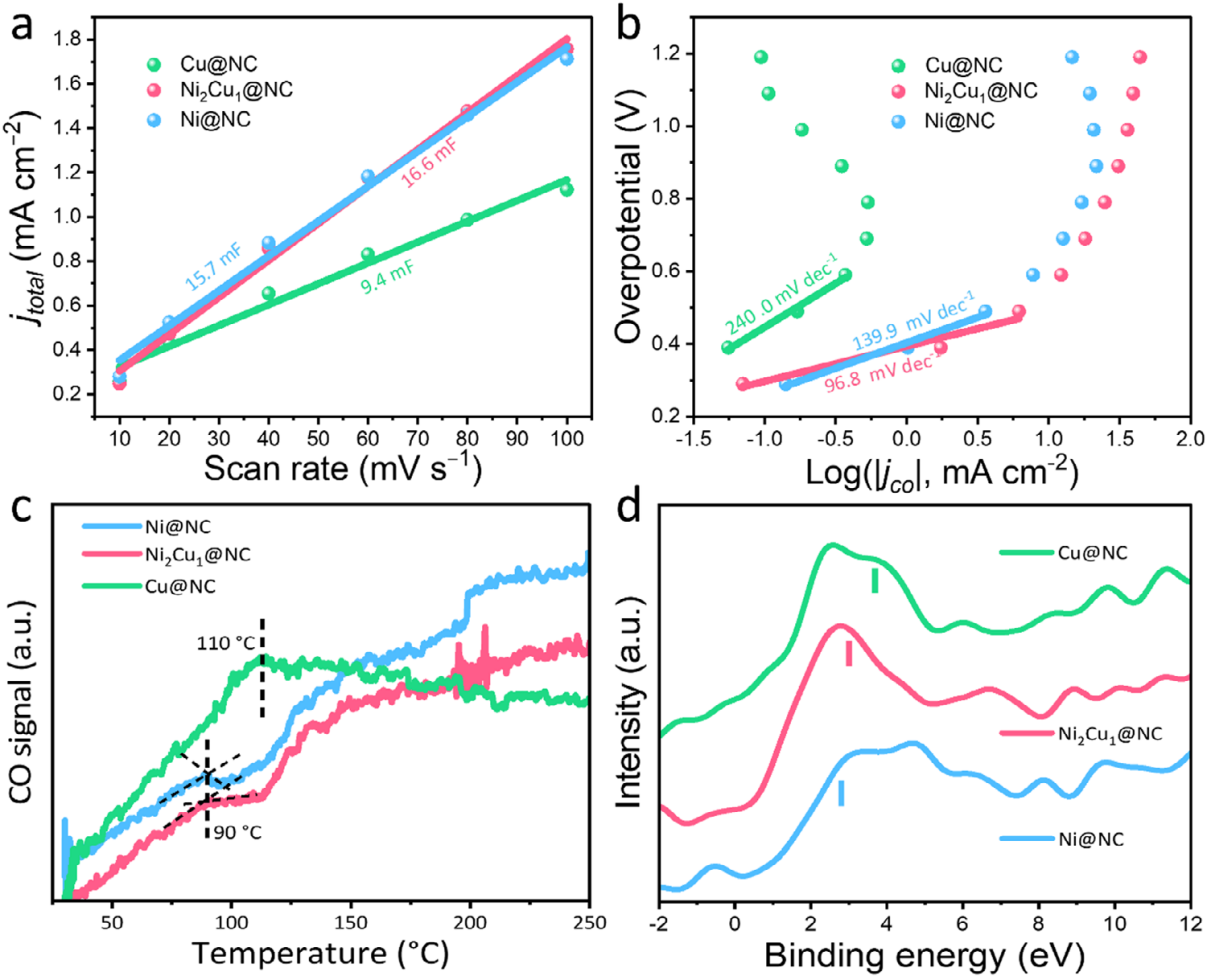
a) Charging current density differences plotted against scan rates, the roughness factor (RF) was calculated from the ratio of ECSA on the electrode to the geometric area of the carbon paper electrode. Roughness factor of Ni@NC, Ni_2_Cu_1_@NC, and Cu@NC electrodes are 830, 785, and 470, respectively; b) Tafel slope for the as‐obtained catalysts; c) CO‐TPD plots of the as‐obtained samples; d) Surface valence band photoemission spectra of Ni@NC, Ni_2_Cu_1_@NC, and Cu@NC relative to the Fermi level. All the spectra are background corrected. The vertical line indicates the *d*‐band center of each sample relative to the Fermi level.

To get more in‐depth insights into the underlying fundamentals of weight ratio‐dependent ECO2RR performances, the electrochemical active surface area (ECSA) was evaluated by measuring double layer capacitance (*C_dl_*) using cyclic voltammetry (CV) at various scan rates (Figure S16, Supporting Information) in a non‐Faradaic potential range of 0.01–0.2 V (vs Ag/AgCl). **Figures** **5**a and S17 (Supporting Information) display the slope of current density against scan rates, which can serve as a reference for the ECSA. Accordingly, the ECSA of Ni@NC, Ni_1_Cu_1_@NC, Ni_2_Cu_1_@NC, Ni_1_Cu_2_@NC, and Cu@NC was determined to be 15.7, 9.3, 16.6, 13.3, and 9.4 mF, respectively. The largest ECSA obtained for Ni_2_Cu_1_@NC discloses the abundant active sites for ECO2RR, which is favorable for the adsorption of the intermediates.^[^ ^72^ ^]^ In addition, the kinetics of the as‐prepared catalysts were evaluated by the Tafel slope. As displayed in Figures 5b and S18 (Supporting Information), Ni_2_Cu_1_@NC demonstrates a much lower Tafel slope of 96.8 mV dec^−1^ than that of Ni@NC, Cu@NC, Ni_1_Cu_1_@NC, and Ni_1_Cu_2_@NC, respectively, suggesting faster reaction kinetics of Ni_1_Cu_2_@NC than its counterparts. Moreover, CO‐TPD was carried out, which can serve as an indicator for estimating the interaction strength of the intermediates on the catalyst surface. As shown in Figure 5c, Cu@NC (110 °C) displays a higher CO desorption temperature than Ni@NC and Ni_2_Cu_1_@NC catalysts (90 °C), suggesting a stronger interaction on the catalyst's surface. The weak CO signals observed for both Ni₂Cu₁@NC and Ni@NC suggest that CO can be quickly released from the catalyst surface once generated.^[^ ^73^, ^74^, ^75^ ^]^ Accordingly, Ni@NC and Ni_2_Cu_1_@NC display faster reaction kinetics and deliver a higher CO selectivity due to the weaker adsorption of the *CO intermediate, which is in good consistency with the Tafel slope result. The strong interaction between *CO and Cu@NC is in good agreement with previous reports.^[^ ^76^ ^]^ Considering that the reactivity of Ni‐based alloys relates to the *d*‐band center, the valence band spectra (VBS) of Ni@NC, Ni_2_Cu_1_@NC, and Cu@NC catalysts were determined by high‐resolution XPS. Figure 5d displays the relationship between the *d*‐band center values of the as‐prepared catalysts. The improvement of the electrocatalytic selectivity and activity for the formation of CO from ECO2RR is attributed to the modification of the electronic structure of Ni in the Ni_2_Cu_1_@NC bimetallic catalyst. The *d*‐band center value was determined according to the previously reported method, and the value for Ni_2_Cu_1_@NC is evaluated to be −3.01 eV, corresponding to a negative shift by 0.22 eV to a lower *d*‐band center position compared to that for Ni@NC (−2.79 eV).^[^ ^77^ ^]^ Density functional theory (DFT) calculations have demonstrated that a downshifted *d*‐band center position is beneficial for electrochemical CO_2_ reduction.^[^ ^78^, ^79^, ^80^ ^]^ According to the *d*‐band theory, specifically, a lower *d*‐band center position usually results in weaker interaction with the adsorbate on catalysts due to the occupancy of antibonding states.^[^ ^81^ ^]^ Accordingly, the enhanced selectivity for CO formation on the Ni_2_Cu_1_@NC can be explained by the downshift of the *d*‐band center.^[^ ^26^, ^34^, ^82^ ^]^ Although Cu@NC is dominated by Cu(111) facet and possesses a much lower *d*‐band position, it displays poor selectivity for the formation of CO, and neither C_1_ nor C_2+_ products were detected, which is attributed to the lower specific surface area bind much less *CO content than other samples according to the ECSA analysis.

## 3 Conclusion

In summary, with the product selectivity of the catalysts related to the electronic structure, we have prepared a series of Ni*_x_* Cu*_y_* @NC bimetallic catalysts with varied Ni to Cu weight ratios. We systematically demonstrated their electrocatalytic selectivity toward the CO formation, of which the high catalytic activity is attributed to the synergistic performance between different metallic nuclei. When evaluated as the catalyst for electrochemical CO_2_ reduction, the Ni_2_Cu_1_@NC bimetallic catalyst displayed an exceptional CO selectivity over 90% in a wide potential range of −0.7 to −1.1 V,  with the maximum FE and partial current density of 94.3% and −24.9 mA cm^−2^ at −0.9 V. The unprecedented performances for the CO formation on Ni_2_Cu_1_@NC are predominantly attributed to the optimal combined Cu content and interactions between Ni and Cu. Specifically, the introduced Cu could result in a lower *d*‐band center position compared to that for Ni@NC, which subsequently leads to a weak adsorption of the *CO intermediate and facilitates the production of CO from Ni_2_Cu_1_@NC. We believe that by systematically constructing, completing, and understanding the catalysts’ map, electrochemical CO_2_ reduction will eventually become a reality.

## 4 Experimental Section

### Chemicals

All the chemicals were used as received. Ni(NO_3_)_2_∙6H_2_O and urea were purchased from Sigma Chemical Company. Melamine was purchased from Sigma–Aldrich. Cu(NO_3_)_2_·3H_2_O and KHCO_3_ were purchased from Acros Organics. Pyromellitic dianhydride (PMDA) was purchased from Alfa Aesar. NH_4_Cl and (NH_4_)_6_Mo_7_O_24_·4H_2_O were purchased from Merck Schuchardt OHG. Ethanol was purchased from VWR International, LLC.

### Synthesis of Bulk g‐C_3_N_4_ Precursor

The g‐C_3_N_4_ was prepared according to the previous report by a thermal polymerization strategy.^[^ ^83^, ^84^ ^]^ In detail, the 10 g melamine was heated from room temperature to 550 °C at the ramping rate of 2.3 °C min^−1^ in a muffle furnace and then maintained at that temperature under an air atmosphere for 4 h. Finally, the light‐yellow bulk g‐C_3_N_4_ was obtained when it cools down to room temperature naturally. The powder g‐C_3_N_4_ was obtained by grinding bulk g‐C_3_N_4_ in a mortar.

### Preparation of NiPPc@g‐C_3_N_4_ and CuPPc@g‐C_3_N_4_

In this research, NiPPc@g‐C_3_N_4_ and CuPPc@g‐C_3_N_4_ were synthesized by modifying the method reported previously.^[^ ^47^ ^]^ In detail, first, 1.0 g of g‐C_3_N_4_ was dispersed in 30 mL of ethanol by stirring. Then, 0.0371 g Ni(NO_3_)_2_∙6H_2_O or 0.285 g Cu(NO_3_)_2_·3H_2_O were dissolved in the above g‐C_3_N_4_ suspension solution. After being kept stirring for 4 h at room temperature, the mixed suspension solution was heated to 80 °C while being kept stirring to remove the solvent. Next, 0.00225 g (NH_4_)_6_Mo_7_O_24_·4H_2_O, 0.2516 g PMDA, 0.5125 g urea, and 0.125 g NH_4_Cl were mixed thoroughly with the as‐obtained Ni^2+^ or Cu^2+^@g‐C_3_N_4_ powder with a pestle mortar. Then, the well‐mixed products were heated to 230 °C at a ramping rate of 3 C min^−1^ under an air atmosphere and kept at 230 °C for 3 h. After cooling down to room temperature, the as‐obtained product was washed with deionized (DI) water and acetone till the solution was transparent. Finally, NiPPc@g‐C_3_N_4_ and CuPPc@g‐C_3_N_4_ precursors were obtained by drying the as‐obtained greenish powder in an oven.

### Preparation of Ni_2_Cu_1_PPc@g‐C_3_N_4_, Ni_1_Cu_1_PPc@g‐C_3_N_4_, and Ni_1_Cu_2_PPc@g‐C_3_N_4_ Precursors

Overall, the Ni_2_Cu_1_PPc@g‐C_3_N_4_, Ni_1_Cu_1_PPc@g‐C_3_N_4_, and Ni_1_Cu_2_PPc@g‐C_3_N_4_ precursors were obtained by following the same procedure as the Ni@g‐C_3_N_4_ and CuPPc@g‐C_3_N_4_ precursors but with varied weight ratios of Ni to Cu (2:1, 1:1, 1:2) based on the metal content in the final product.

### Preparation of Ni@NC, Ni_2_Cu_1_@NC, Ni_1_Cu_1_@NC, Ni_1_Cu_2_@NC, and Cu@NC Catalysts

The Ni@NC, Ni_2_Cu_1_@NC, Ni_1_Cu_1_@NC, Ni_1_Cu_2_@NC, and Cu@NC catalysts were synthesized by carbonizing the corresponding precursors at 800 °C for 2 h under an argon atmosphere with an increase rate of 2 °C min^−1^.

### Materials Characterizations

XRD was employed at the step length of 0.04° on a Bruker D8 equipped with Cu Kα radiation (λ = 1.54 Å). Raman spectra were collected on XploRa (HORIBA) with a laser length of 532 and 785 nm. FTIR spectra were collected on ThermoFisher (Nicolet 6700) at room temperature. The transmission electron microscopy (TEM) and high‐resolution TEM images equipped with EDX were taken on FEI (Talos F200X G2) at an accelerate voltage of 200 kV. Nitrogen adsorption–desorption isotherm measurements were performed by QUADRASORB SI (Quantachrome) at 77 K. X‐ray photoelectron spectroscopy (XPS) measurements were implemented on a Prevac spectrometer equipped with a hemispherical VG SCIENTA R3000 analyzer (pass energy of 100 eV) and a monochromatized aluminum source AlKα (1486.6 eV). The binding energy was calibrated using the C 1s peak located at 284.8 eV. The spectrum fitting was conducted using the Casa XPS software with Gaussian–Lorentzian peak shapes after subtraction of Shirley background. VB‐XPS measurement was carried out on Thermo Scientific K‐Alpha with a step length of 0.1 eV. The valence band center was determined by a numerical integration in the range of 10−0 eV and placing the center at the mean value of the integrated area, which is similar to the previously reported method.^[^ ^77^ ^]^

### ICP‐Measurements

The mass content of Ni and Cu in the composite was determined by ICP‐MS (Aligent 7500 Series). First, 5 mg as‐obtained catalyst was destroyed in 150 µL HNO_3_ (Traceselect) and 350 µL HCl (Traceselect) mixed solution at 70 °C overnight. Then the mixed solution was diluted to a total volume of 10 mL. After that, 100 µL destructed solution was then further diluted to 10 mL after adding 150 µL of HNO_3_. A final dilution was made containing 100 µL of the ×100 solution, 100 µL of 10 ppm Y (internal standard), 150 µL HNO_3_, and diluted to 10 mL. 1 ppm standards were made by diluting a 1000 ppm Stock (Alfa Aesar) with HNO_3_ and Y.^[^ ^85^ ^]^

### Electrochemical Measurements

All the electrochemical measurements were carried out in a gas‐tight H‐cell that was separated by a cation exchange membrane (Nafion 117). All the signal was recorded by Autolab PGSTAT101. A platinum electrode (1 cm^2^) was used as the counter electrode. An Ag/AgCl (3 m KCl) was served as the reference electrode. All the potentials reported in this work were converted to versus RHE according to the equation otherwise stated.

(1) $$V_{\mathrm{RHE}}=V_{\mathrm{Ag}/\mathrm{AgCl}}+0.21+0.0592*\mathrm{pH}$$

The pH value of the CO_2_ saturated 0.5 m KHCO_3_ is determined to be 7.2. The linear cyclic voltammetry (LSV) was carried out at a scan rate of 10 mV s^−1^ after activation by cyclic voltammetry for 20 cycles at a scan rate of 100 mV s^−1^. The electrochemical active surface area (ECSA) of the obtained catalysts was evaluated by measuring their double layer capacitance at various scan rates (*v*) in a potential range of 0.01–0.2 V versus Ag/AgCl. Roughness factor (RF) was calculated according to the ratio of the ECSA on the electrode to the geometric area of the carbon paper electrode based on Equation (2).

(2) $$\mathrm{ECSA}=C_{\mathrm{dl}}/C_{s}$$

where *C_dl_* corresponds to the slope of the double‐layer charging current versus the scan rate (*ν*) plot, a specific capacitance (*C_s_*) value of 20 µF cm^−2^ was used. All data are reported in this work without iR compensation.

The liquid product was analyzed by ^1^H nuclear magnetic resonance (NMR) with a Bruker Avance III 400 MHz spectrometer. The gas products were determined by gas chromatography (GC, Thermal‐Fisher 1310) equipped with a flame ionization detector (FID) and thermal conductivity detector (TCD). Faradic efficiency (FE) of CO was obtained by Equation 3.

(3) $$\mathrm{FE}=\frac{Z_{i}\times V_{i}\times G\times F\times P_{0}}{I\times R\times T\times 60\;000}$$

where *Z_i_* indicates the number of electrons required to produce an *i* molecule, here, *i* = 2 for CO and H_2_; *V_i_* is the volume ratio of product *i* in the GC sampling loop; G suggests the flow rate of CO_2_ during the test; *F* is the Faradaic constant (96 485 C mol^−1^); *P_o_* represents the atmospheric pressure (1.013 × 10^5^ Pa); *I* represents the average current in a period (t) of electrocatalysis; *R* is the ideal gas constant (8.314 J mol^−1^ K^−1^); *T_0_* is the reaction temperature (298 K).

## Conflict of Interest

The authors declare no conflict of interest.

## Supporting information



Supporting Information

## Data Availability

The data that support the findings of this study are available in the supplementary material of this article.
