# Supplementary material for: Strategic Modulation of CO Intermediate Desorption Dynamics on Bimetallic NixCuy@NC Catalyst: Synergistic Electrocatalysis for Sustainable CO2 Conversion
Source: Small. 2025 Jun 6;21(31):2505306. doi: 10.1002/smll.202505306 (PMC12332811; doi:10.1002/smll.202505306)
Supplement: Supplementary file 1 — Supporting Information [file SMLL-21-2505306-s001.docx]

Supporting Information

**Strategic Modulation of CO Intermediate Desorption Dynamics on Bimetallic Ni_x_Cu_y_@NC Catalyst: Synergistic Electrocatalysis for Sustainable CO_2_ Conversion**

*Jian Zhu, Guangchao Li, Anna Rokicińska, Zhenyu Wang, Piotr Kuśtrowski, Zhouguang Lu, Shoubhik Das^*^, Pegie Cool^*^*

J. Zhu, S. Das, and P. Cool

Department of Chemistry

University of Antwerp

Antwerp 2610, Belgium

E-mail: Pegie.Cool@uantwerpen.be

S. Das

Department of Chemistry

University of Bayreuth

Bayreuth 95447, Germany

E-mail: Shoubhik.Das@uni-bayreuth.de

G. Li

School of Metallurgy and Environment

Central South University

Changsha 410083, China
A. Rokicińska and P. Kuśtrowski

Faculty of Chemistry

Jagiellonian University

Kraków 30-387, Poland

Z. Wang and Z. Lu

Department of Materials Science and Engineering

Southern University of Science and Technology

Shenzhen 518055, China

**
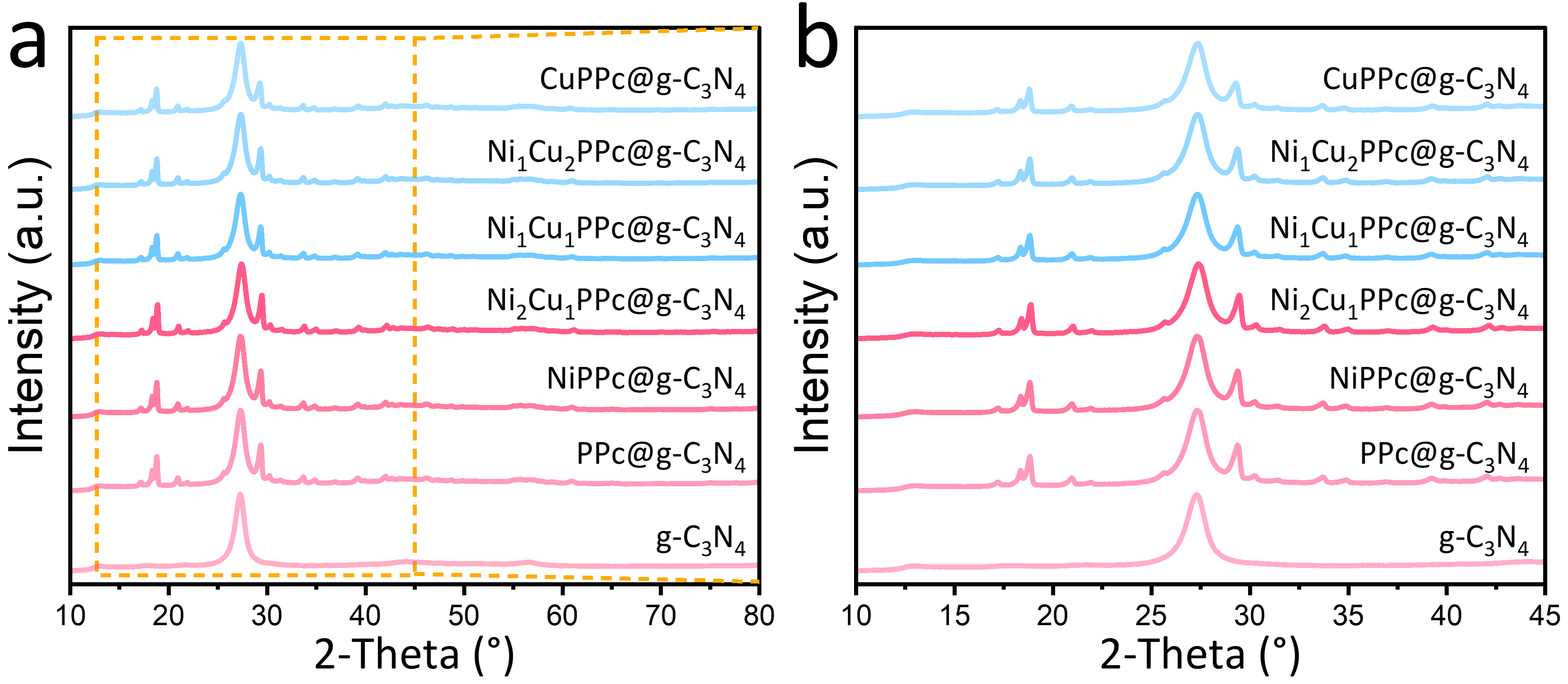
**

**Figure S1.** a) XRD pattern of the Ni*_x_*Cu*_y_*PPc@g-C_3_N_4_, b) enlarged XRD pattern of the as-prepared Ni*_x_*Cu*_y_*PPc@g-C_3_N_4_ in Figure S1a.

**
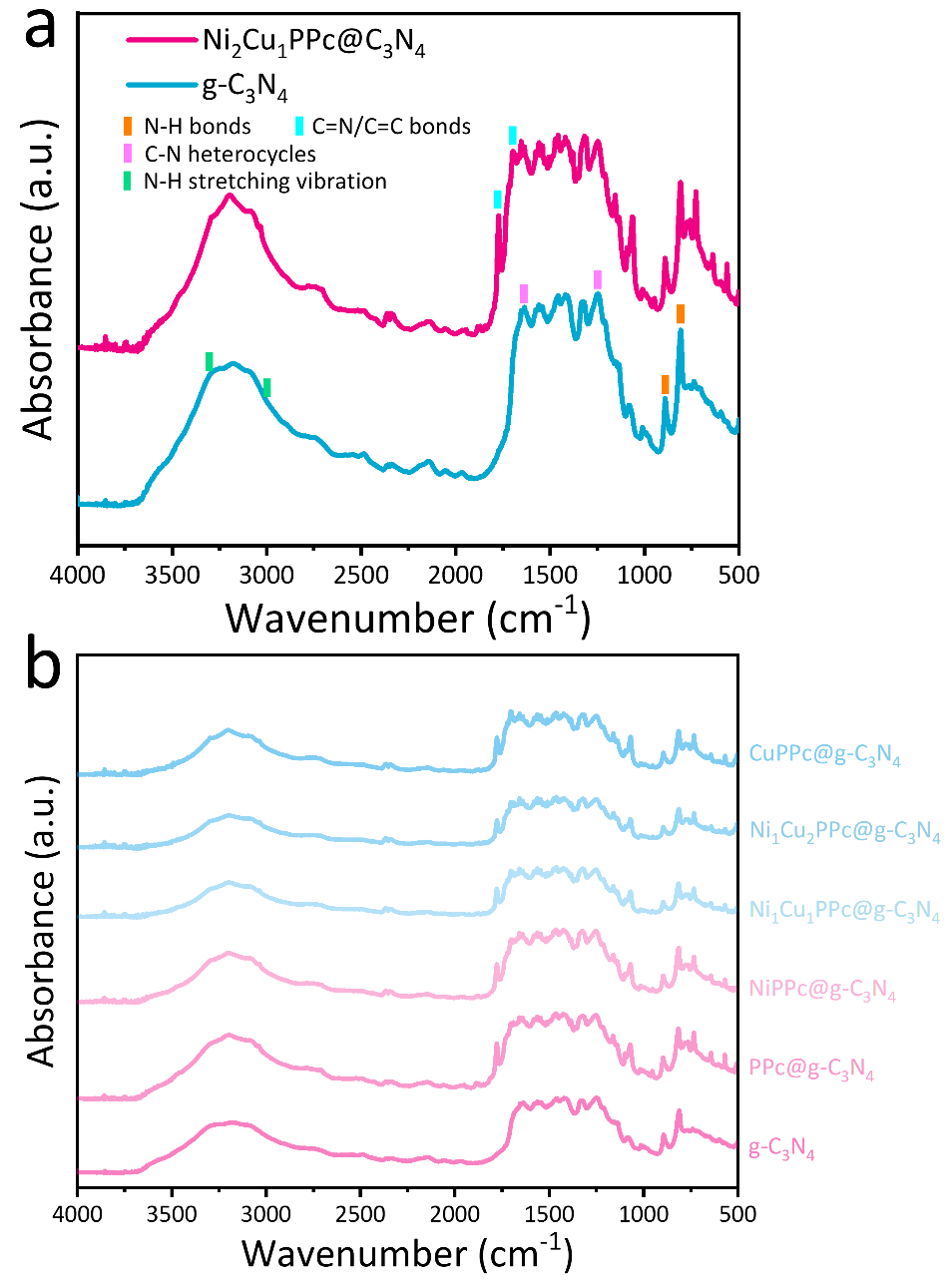
**

**Figure S2.** a) FTIR spectra of Ni_2_Cu_1_PPc@g-C_3_N_4_ and g-C_3_N_4_, b) FTIR spectra of the as-prepared samples.

**
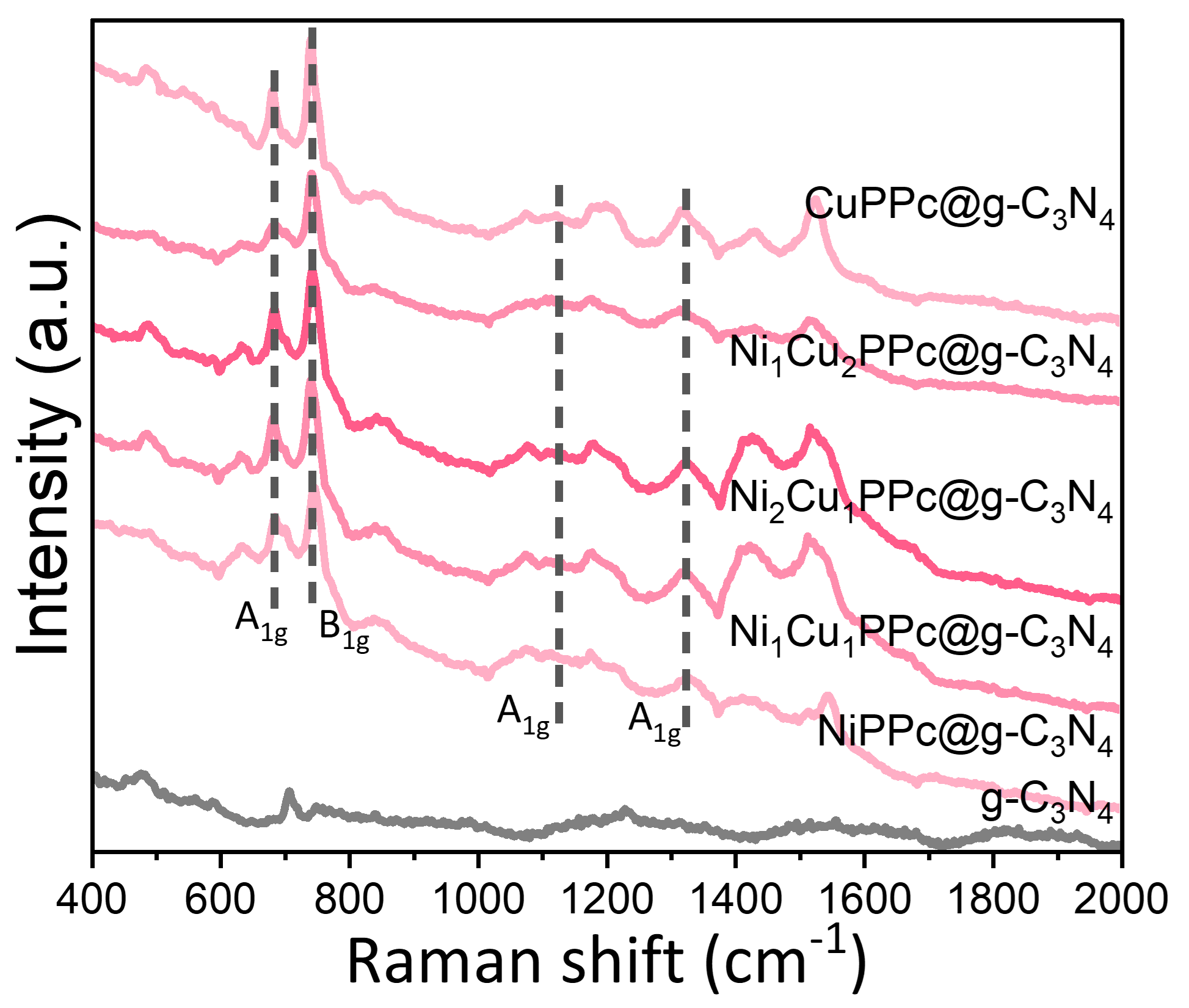
**

**Figure S3.** Raman spectra of the as-obtained samples.

**
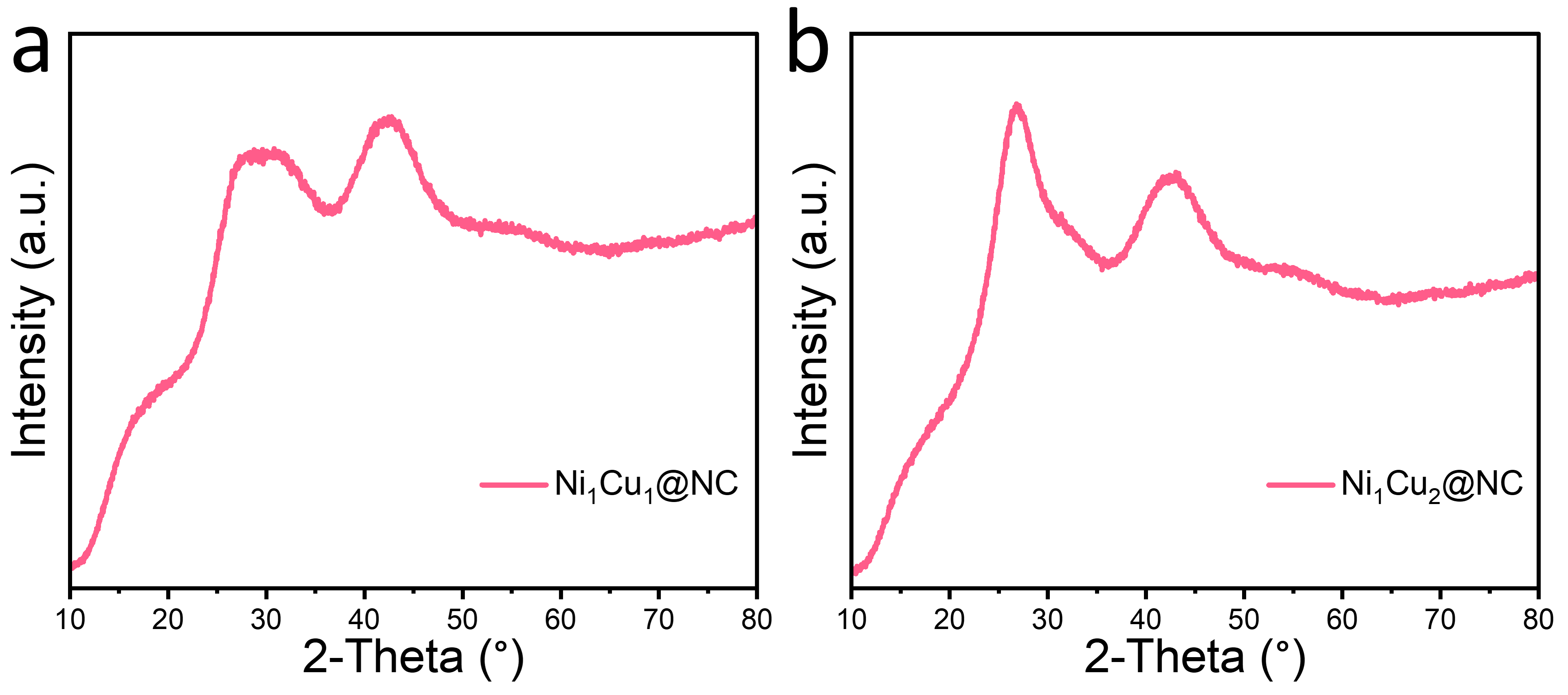
**

**Figure S4.** XRD pattern of a) Ni_1_Cu_1_@NC, b) Ni_1_Cu_2_@NC.

**Figure S5.** Particle size distribution of nanoalloys supported on graphene layers.


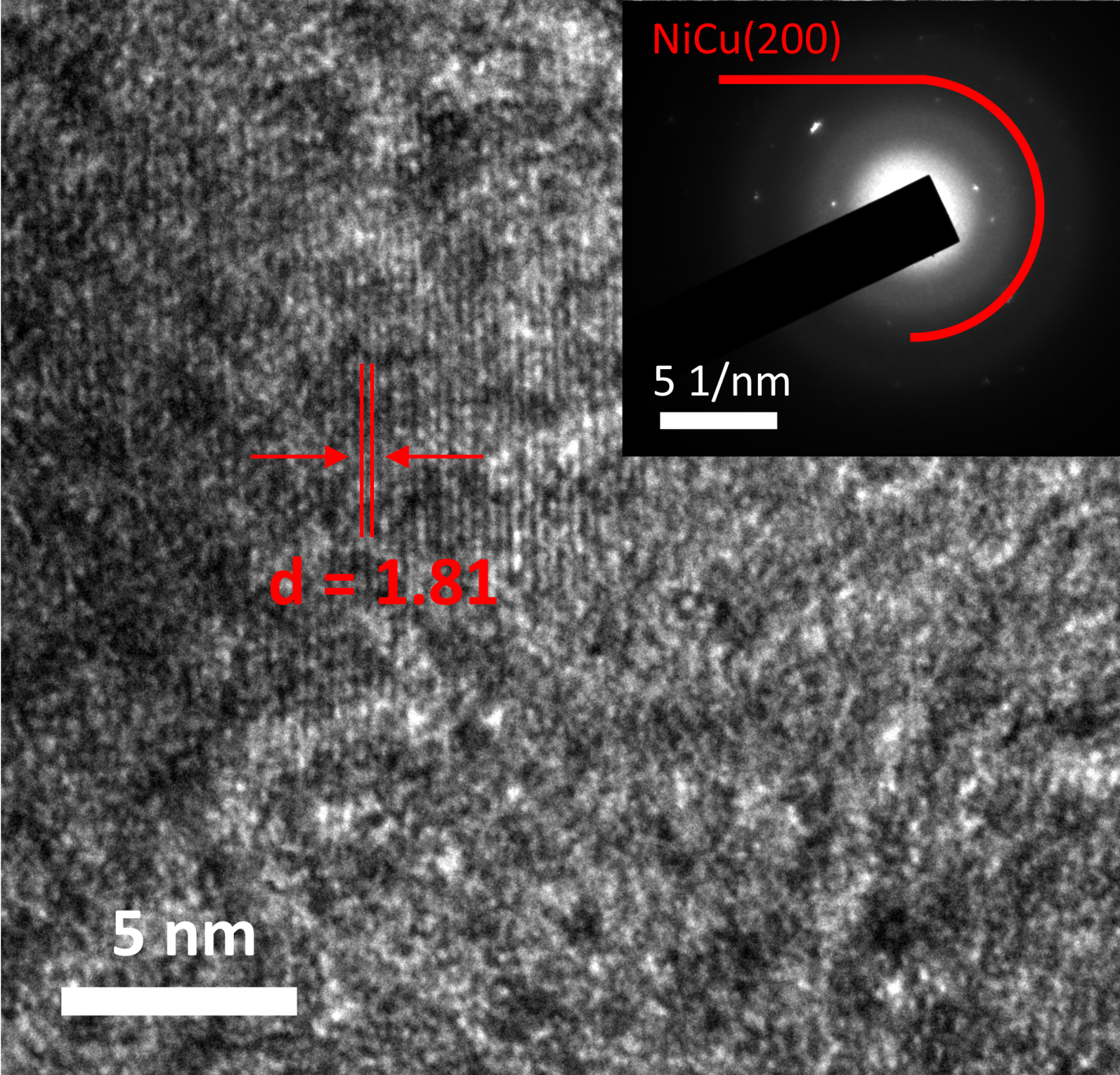


**Figure S6.** High-resolution TEM image of Ni₂Cu₁@NC. *Inset*: Selected area electron diffraction (SAED) pattern of the Ni₂Cu₁@NC catalyst.

**
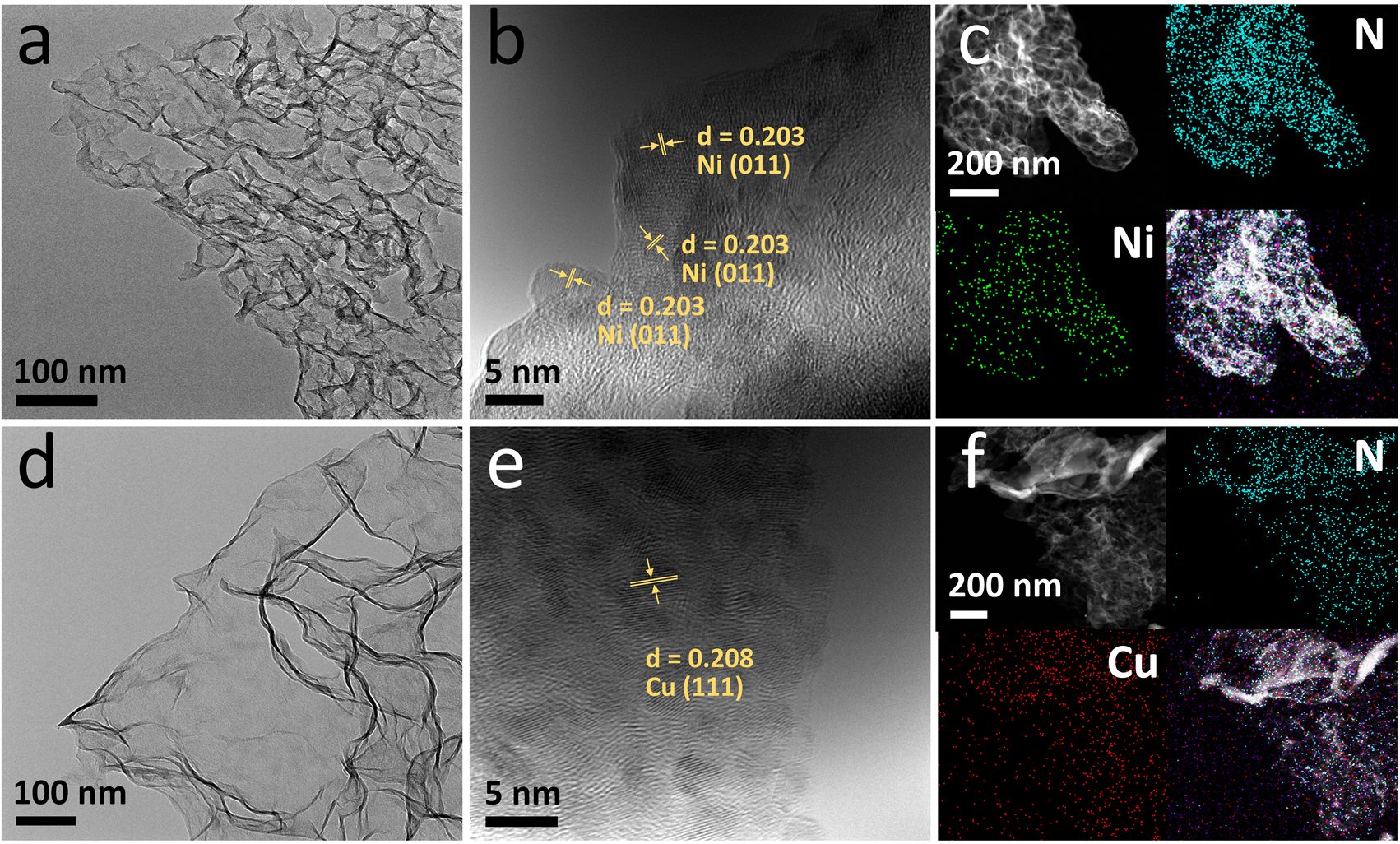
**

**Figure S7.** a) and d) TEM images, b) and e) High-resolution image, c) and f) elemental mapping of Ni@NC and Cu@NC.

**
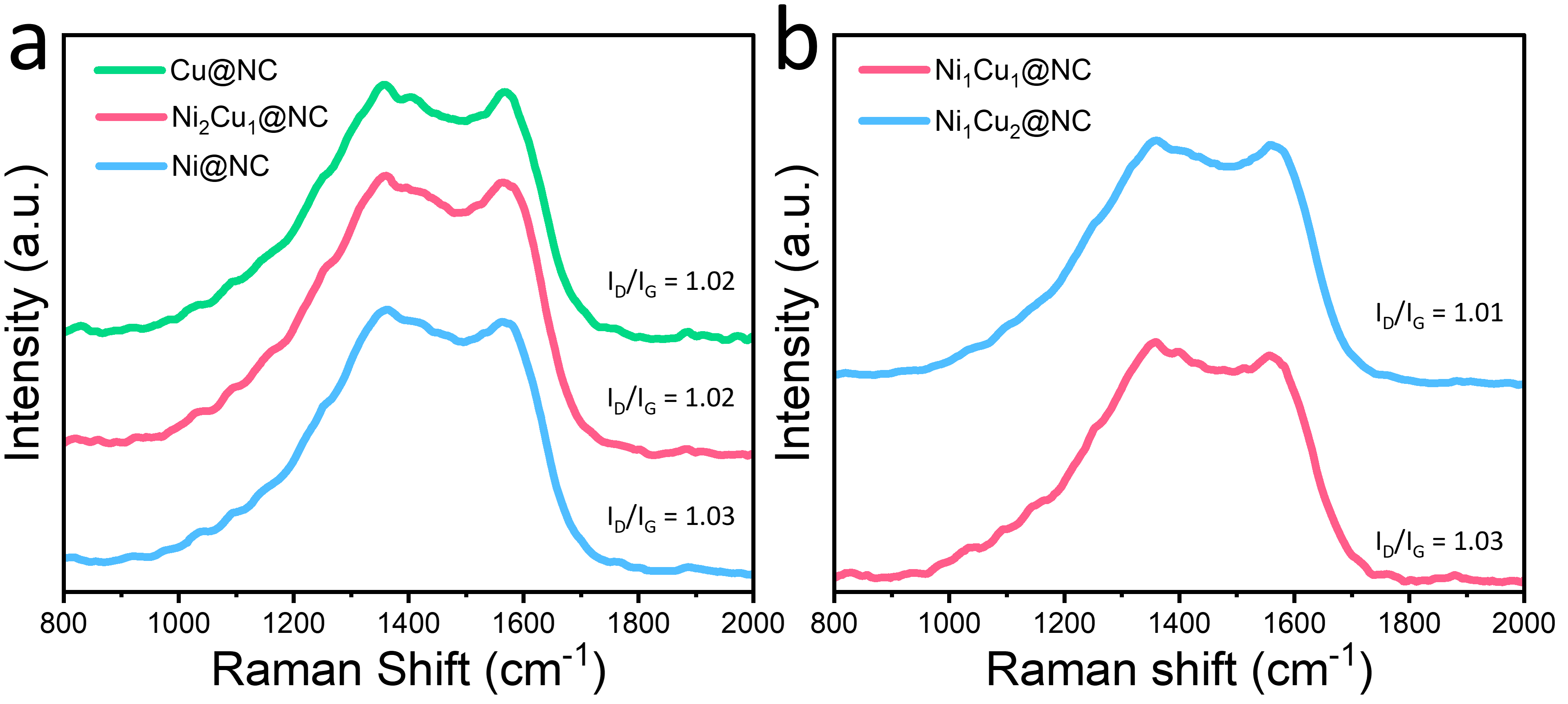
**

**Figure S8.** Raman spectra of the as-prepared samples.

**
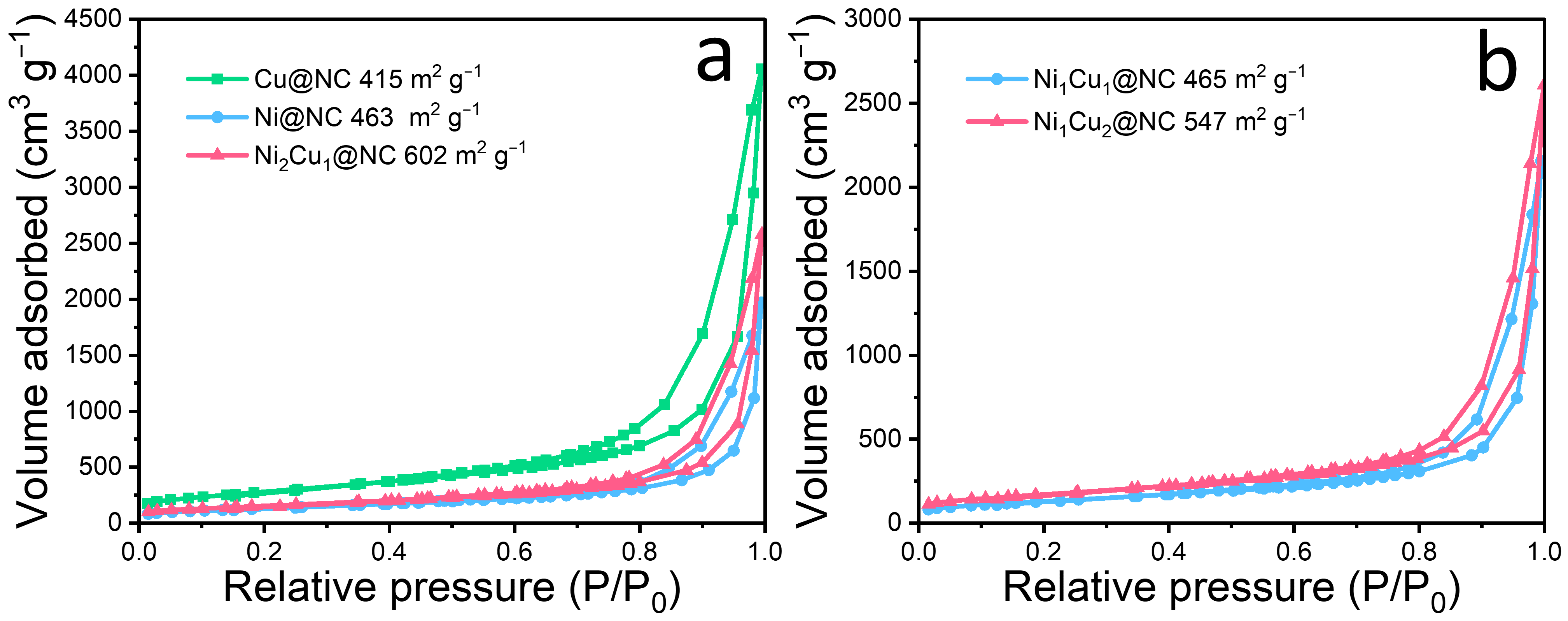
**

**Figure S9.** N_2_ adsorption-desorption isotherms of the as-synthesized catalysts.


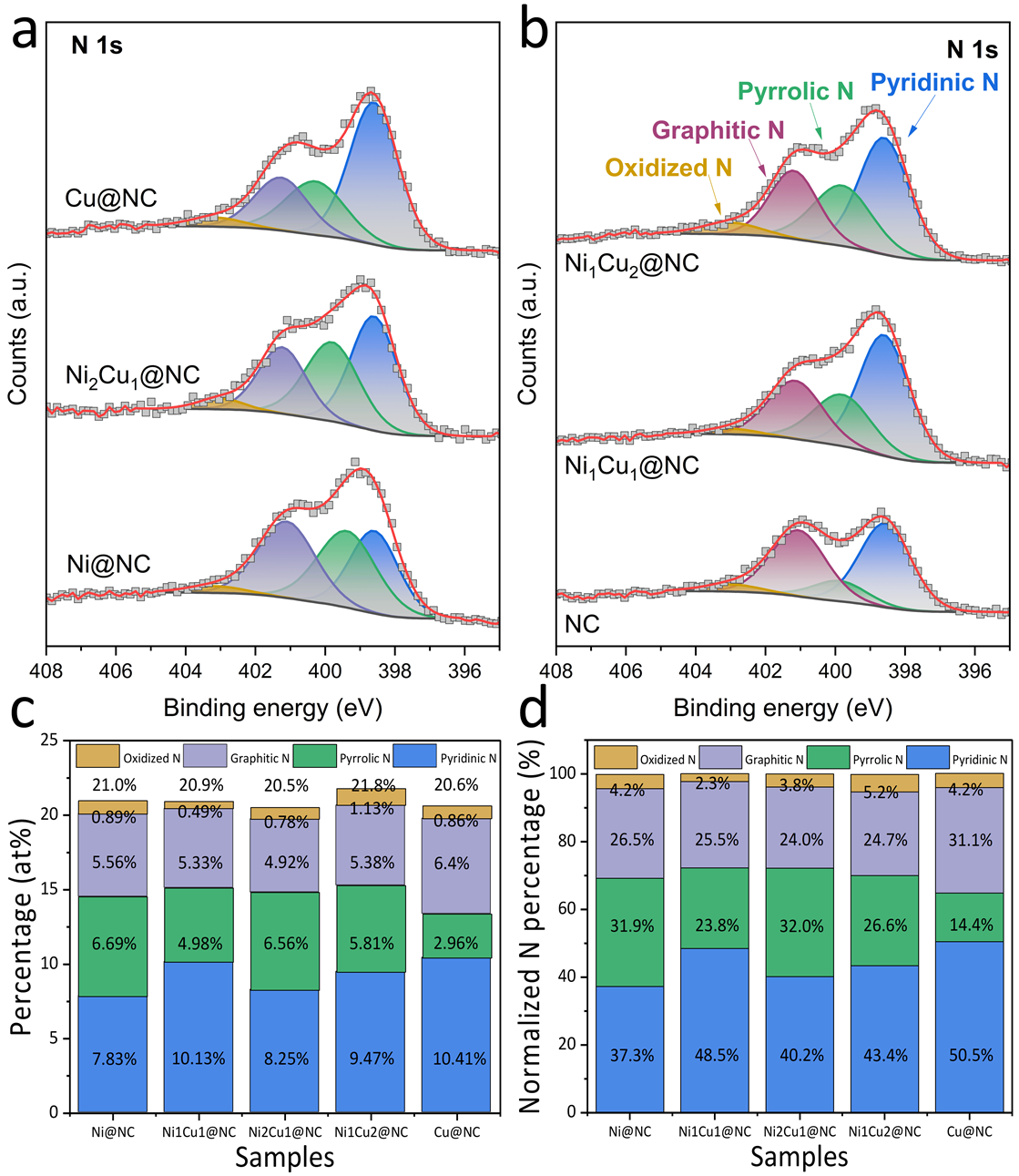


**Figure S10.** a) and b) High-resolution N 1s XPS spectra of the as-prepared samples, c) and d) Calculated N content of the as-synthesized samples.

**Figure S11.** LSV curves of Ni_1_Cu_1_@NC and Ni_1_Cu_2_@NC in an Ar and N_2_ saturated 0.5 M electrolyte, respectively.

**
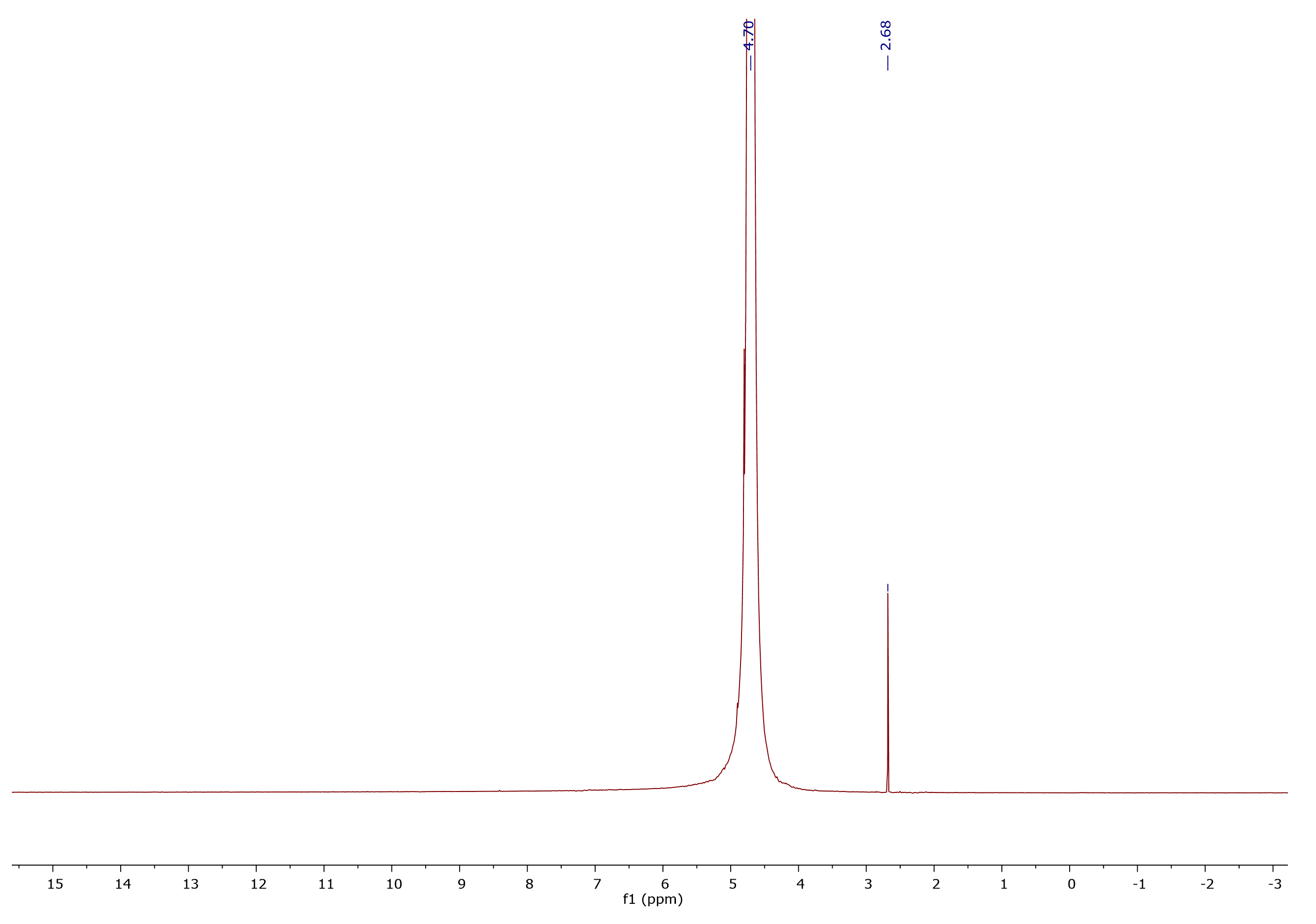
**

**Figure S12.** ^1^H NMR of the 0.5 M KHCO_3_ electrolyte after electrolysis.

**Figure S13.** CO Faradaic efficiency of Ni_1_Cu_1_@NC and Ni_1_Cu_2_@NC at various potentials.

**Figure S14.** Partial current density of Ni_1_Cu_1_@NC and Ni_1_Cu_2_@NC at various potentials.

**Figure S15.** Electrocatalytic stability test at −0.9 V in a CO_2_-saturated 0.5 M KHCO_3_ electrolyte.


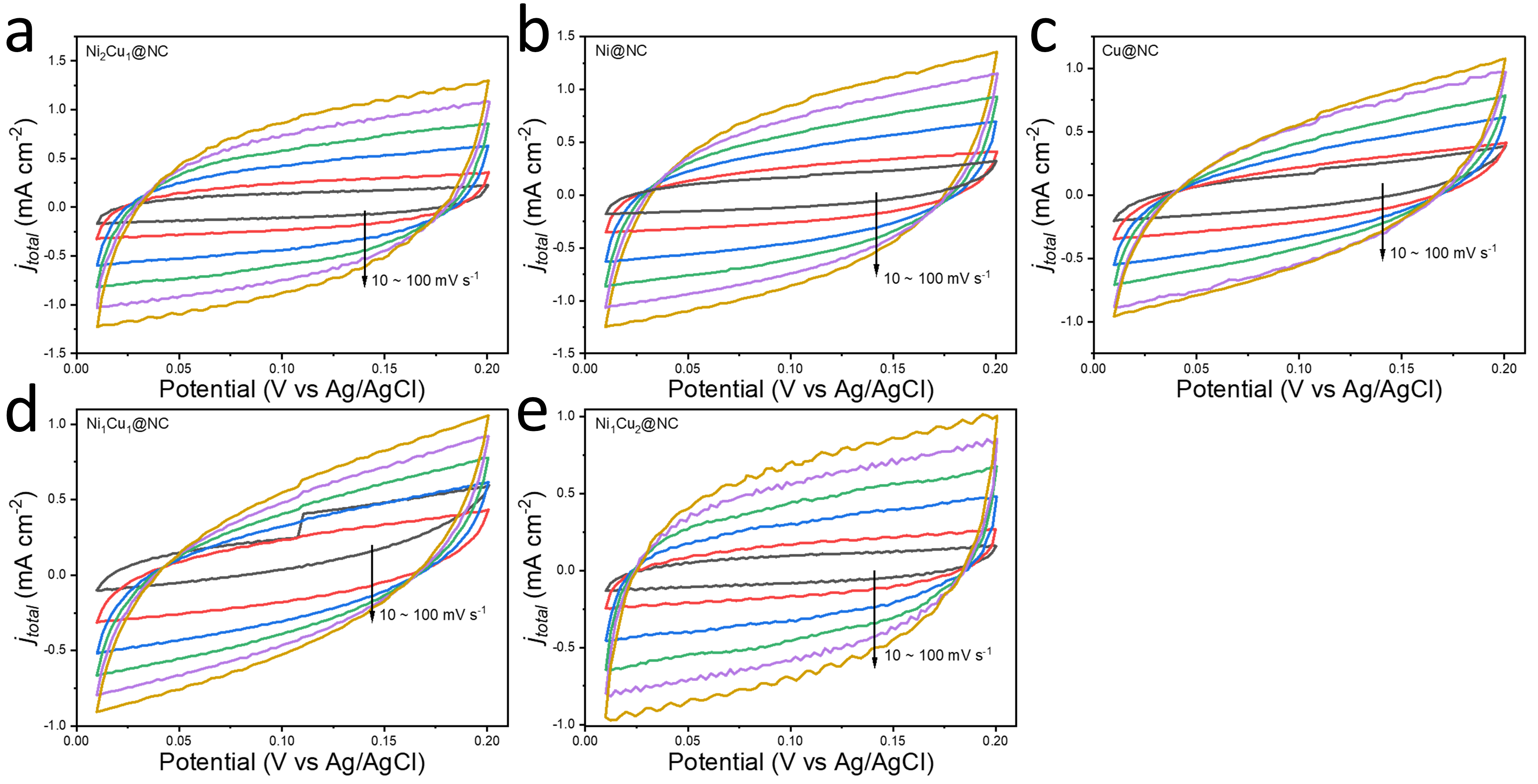


**Figure S16.** Cyclic voltammetry (CV) curves of the as-prepared samples at various scan rate in the potential range of 0.01 −0.2 V (vs. Ag/AgCl).


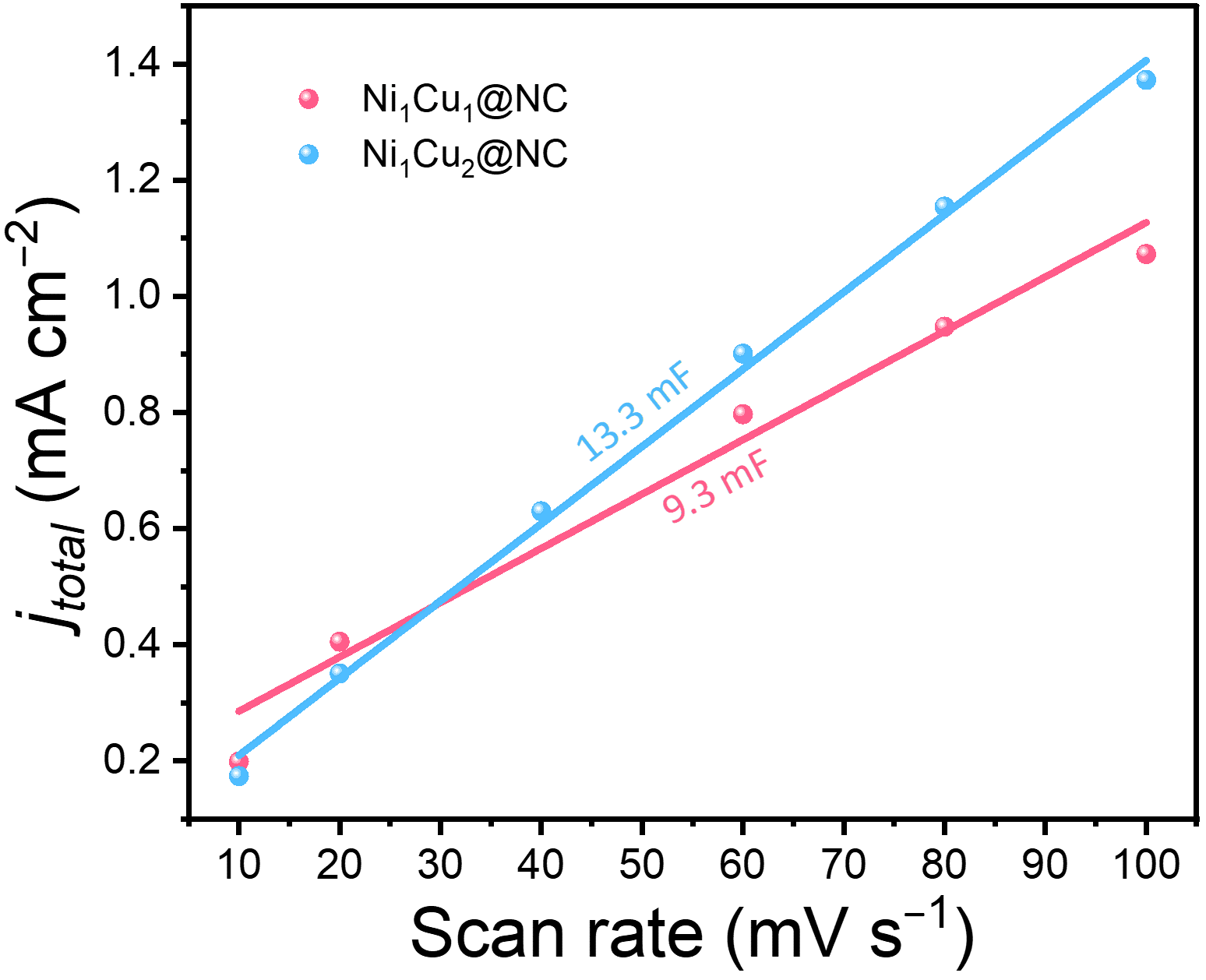


**Figure S17.** Charging current density differences plotted against scan rates, roughness factor (RF) was calculated from the ratio of ECSA on the electrode to the geometric area of the carbon paper electrode. Roughness factor of Ni_1_Cu_1_@NC and Ni_1_Cu_2_@NC are 665 and 465, respectively.


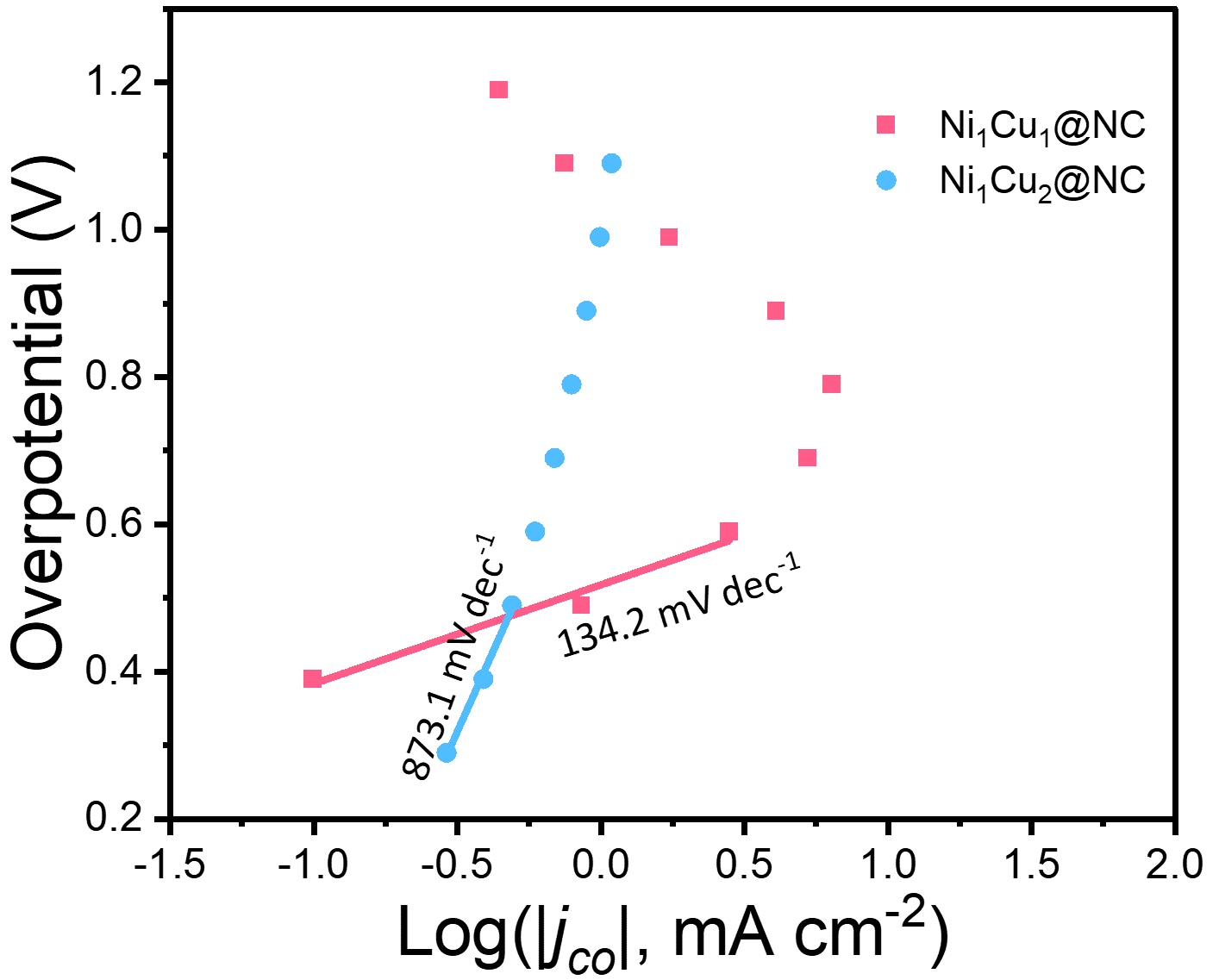


**Figure S18.** Tafel slope of the as-obtained Ni_1_Cu_1_@NC and Ni_1_Cu_2_@NC samples.

**Table S1.** Ni and Cu content of the as-prepared samples according to the ICP-MS measurement.

| **Sample name** | **Ni (wt%)** | **Cu (wt%)** | **Total (wt%)** |
| --- | --- | --- | --- |
| Ni@NC | 8.92 | 0 | 8.92 |
| Ni_1_Cu_1_@NC | 4.59 | 5.48 | 10.07 |
| Ni_2_Cu_1_@NC | 5.88 | 3.39 | 9.27 |
| Ni_1_Cu_2_@NC | 3.05 | 6.96 | 10.01 |
| Cu@NC | 0 | 9.44 | 9.44 |

**Table S2.** Ni and Cu contents on the as-prepared samples’ surface determined by XPS measurements.

| **Sample name** | **Ni^2+^ (at%)** | **Cu^+^ (at%)** | **Cu^2+^ (at%)** |
| --- | --- | --- | --- |
| Ni@NC | 3.9 | - | - |
| Ni_1_Cu_1_@NC | 2.4 | 3.1 | 0.6 |
| Ni_2_Cu_1_@NC | 3.3 | 3.0 | 0.0 |
| Ni_1_Cu_2_@NC | 0.9 | 2.0 | 0.4 |
| Cu@NC | - | 4.7 | 1.8 |

**Table S3.** Comparison of ECO2RR performances with recently reported advanced catalysts in H-type cell.

| **Catalyst** | **Main product** | **FE at V (vs RHE)** | ***j_CO_*** | **FE > 90%** | **Ref.** |
| --- | --- | --- | --- | --- | --- |
| [Ni_2_Cu_1_@NC](mailto:Ni2Cu1@NC) | CO | 94.3% at −0.9 V | −44 mA cm^−2^ at −1.4 V | −0.7 ~ −1.1 V | **This work** |
| Co-N-Ni/NPCNSs | CO | 96.4% at −0.48 V | −12.9 mA cm^−2^ at −0.74 V | −0.48 ~ −0.68 V | 1 |
| Pd_85_Cu_15_/C | CO | 86% at −0.89 V | −5.9 mA cm^−2^ at −0.89 V | no | 2 |
| Pd_5_@Au_95_ | CO | 80% at −0.5 V | −1.6 mA cm^−2^ at −0.5 V | no | 3 |
| C-Fe-Co-ZIF-1.6wt%-Fe | CO | 52% at −0.55 V | −8.0 mA cm^−2^ at −0.7 | no | 4 |
| Cu_0.18_Cd_0.5_-O | CO | 88.7% at −0.86 V | −27.9 mA cm^−2^ at −1.16 V | no | 5 |
| Ag_88_Cu_12_ | CO | 89.4% at −0.89 V | −5.86 mA cm^−2^ at -0.89 V | −0.9 V | 6 |
| s-PdNi/CNFs-1000 | CO | 96.6% at −0.88 V | −12.0 mA cm^−2^ at −0.88 V | −0.78 ~ −1.18 V | 7 |
| ZIF-NC-Ni-Fe | CO | 97.8% at −0.6 V | −18.6 mA cm^−2^ at −0.9 V | −0.3 ~ −1 V | 8 |
| Ni-Fe-NC | CO | 97% at −0.7 V | −7.4 mA cm^−2^ at −0.7 V | −0.4 ~ −0.9 V | 9 |
| Cu-S-Ni/SNC | CO | 98.1% at −0.65 V | ~ −35 mA cm^−2^ at −0.95 V | −0.65 ~ −0.85 V | 10 |
| InNi DS/NC | CO | 96.7 % at −0.7 V | −23.5 mA cm^−2^ at −1.0 V | −0.5 ~ −0.8 V | 11 |
| Fe/Se-N-C | CO | 95.6 % at −0.45 V | −13.1 mA cm^−2^ at −0.9 V | −0.4 ~ −0.5 V | 12 |
| Ag_76_Sn_24_ | Formate | 87.2% at −0.9 V | −25.4 mA cm^−2^ at −1.06 V | no | 13 |
| In/Bi-750 | Formate | 97.17% at −1.0 V | −32.22 mA cm^−2^ at −1.4 V | −0.8 ~ −1.4 V | 14 |
| Bi_5_Sn_60_ | Formate | 94.8% at −1.0 V | −34 mA cm^−2^ at −1.0 V | −1.0 V | 15 |
| CuBi-100 | Formate | 94.7% at −1.0 V | −12.8 mA cm^−2^ at −1.2 V | −0.9 ~ −1.2 V | 16 |
| Bi-Cu (2:1) | Formate | 94.1% at −1.0 V | −13.5 mA cm^−2^ at −1.0 V | −0.8 ~ −1.2 V | 17 |
| Cu_1_Sn_3_-CC | Formate | 91.38% at −0.8 V | −12.6 mA cm^−2^ at −0.8 V | -0.8 V | 18 |
| Cu_1_Bi_1_ | Formate | 98.07% at −0.98 V | −56.12 mA cm^−2^ at −1.28 V | −0.78 ~ -1.18 V | 19 |
| Cu/Au | Formate | 81% at −0.6 V | −0.67 mA cm^−2^ at −0.6 V | no | 20 |
| Bi-Sn | Formate | 93.9% at −1.0 V | −9.3 mA cm^−2^ at −1.0 V | −0.9 ~ −1.2 V | 21 |
| Ni/Cu-PASC | C_2+_ | 55% at −0.8 V | −4.5 mA cm^−2^ at −0.8 V | no | 22 |
| TWN-Cu_13.35_-600-SACs | C_2+_ | 81.9% at −1.1 V | −35 mA cm^−2^ at −1.1 V | no | 23 |
| Cu_98_Pd_2_ | C_2+_ | 75.6% at −1.15 V | −225 mA cm^−2^ at −1.32 V | no | 24 |
| o-ZSO | C_1_ | 71.88% at −1.1 V | −7.31 mA cm^−2^ at −1.1 V | no | 25 |
| Cu_3_Pd | C_1_ | 40.6% at −1.2 V | < −5 mA cm^−2^ at −1.2 V | no | 26 |

**References**

[1] J. Pei; T. Wang; R. Sui; X. Zhang; D. Zhou; F. Qin; X. Zhao; Q. Liu; W. Yan; J. Dong; et al. N-Bridged Co–N–Ni: new bimetallic sites for promoting electrochemical CO_2_ reduction. *Energy Environ. Sci.* **2021**, *14* (5), 3019-3028.

[2] Z. Yin; D. Gao; S. Yao; B. Zhao; F. Cai; L. Lin; P. Tang; P. Zhai; G. Wang; D. Ma; et al. Highly selective palladium-copper bimetallic electrocatalysts for the electrochemical reduction of CO_2_ to CO. *Nano Energy* **2016**, *27*, 35-43.

[3] Y. Wang; L. Cao; N. J. Libretto; X. Li; C. Li; Y. Wan; C. He; J. Lee; J. Gregg; H. Zong; et al. Ensemble Effect in Bimetallic Electrocatalysts for CO_2_ Reduction. *J. Am. Chem. Soc.* **2019**, *141* (42), 16635-16642.

[4] Z. Chen; G. Zhang; Y. Wen; N. Chen; W. Chen; T. Regier; J. Dynes; Y. Zheng; S. Sun. Atomically Dispersed Fe-Co Bimetallic Catalysts for the Promoted Electroreduction of Carbon Dioxide. *Nano-Micro Lett.* **2021**, *14* (1), 25.

[5] C. Wang; X. Hu; B. Chen; H. Ren; X. Wang; Y. Zhang; X. Chen; Y. Liu; Q. Guan; W. Li. Performance enhancement and active sites identification of Cu-Cd bimetallic oxide derived catalysts for electrochemical CO_2_ reduction. *J. Energy Chem.* **2024**, *91*, 50-58.

[6] W. Wang; S. Gong; J. Liu; Y. Ge; J. Wang; X. Lv. Ag-Cu aerogel for electrochemical CO_2_ conversion to CO. *J. Colloid Interface Sci.* **2021**, *595*, 159-167.

[7] J. Hao; Z. Zhuang; J. Hao; K. Cao; Y. Hu; W. Wu; S. Lu; C. Wang; N. Zhang; D. Wang; et al. Strain Relaxation in Metal Alloy Catalysts Steers the Product Selectivity of Electrocatalytic CO_2_ Reduction. *ACS Nano* **2022**, *16* (2), 3251-3263.

[8] Y. Li; W. Shan; M. J. Zachman; M. Wang; S. Hwang; H. Tabassum; J. Yang; X. Yang; S. Karakalos; Z. Feng; et al. Atomically Dispersed Dual-Metal Site Catalysts for Enhanced CO_2_ Reduction: Mechanistic Insight into Active Site Structures. *Angew. Chem.-Int. Ed.* **2022**, *61* (28), e202205632.

[9] W. Ren; X. Tan; W. Yang; C. Jia; S. Xu; K. Wang; S. C. Smith; C. Zhao. Isolated Diatomic Ni-Fe Metal-Nitrogen Sites for Synergistic Electroreduction of CO_2_. *Angew. Chem.-Int. Ed.* **2019**, *58* (21), 6972-6976.

[10] Z. Sun; C. Li; Z. Wei; F. Zhang; Z. Deng; K. Zhou; Y. Wang; J. Guo; J. Yang; Z. Xiang; et al. Sulfur-Bridged Asymmetric CuNi Bimetallic Atom Sites for CO_2_ Reduction with High Efficiency. *Adv. Mater.* **2024**, *36* (33), e2404665.

[11] Z. Fan; R. Luo; Y. Zhang; B. Zhang; P. Zhai; Y. Zhang; C. Wang; J. Gao; W. Zhou; L. Sun; et al. Oxygen-Bridged Indium-Nickel Atomic Pair as Dual-Metal Active Sites Enabling Synergistic Electrocatalytic CO_2_ Reduction. *Angew. Chem.-Int. Ed.* **2023**, *62* (7), e202216326.

[12] Z. Li; Z. Zhu; J. Wang; Y. Lin; W. Li; Y. Chen; X. Niu; X. Qi; J. Wang; J. S. Chen; et al. Asymmetric Coordination of Heterogeneous Fe‐Se Dual‐atom Sites Boosts CO_2_ Electroreduction. *Adv. Funct. Mater.* **2024**, 2410552.

[13] W. Luc; C. Collins; S. Wang; H. Xin; K. He; Y. Kang; F. Jiao. Ag-Sn Bimetallic Catalyst with a Core-Shell Structure for CO_2_ Reduction. *J. Am. Chem. Soc.* **2017**, *139* (5), 1885-1893.

[14] Q. Wang; X. Yang; H. Zang; C. Liu; J. Wang; N. Yu; L. Kuai; Q. Qin; B. Geng. InBi Bimetallic Sites for Efficient Electrochemical Reduction of CO_2_ to HCOOH. *Small* **2023**, *19* (41), e2303172.

[15] Z. Li; Y. Feng; Y. Li; X. Chen; N. Li; W. He; J. Liu. Fabrication of Bi/Sn bimetallic electrode for high-performance electrochemical reduction of carbon dioxide to formate. *Chem. Eng. J.* **2022**, *428*, 130901.

[16] Y. Xiong; B. Wei; M. Wu; B. Hu; F. Zhu; J. Hao; W. Shi. Rapid synthesis of amorphous bimetallic copper-bismuth electrocatalysts for efficient electrochemical CO_2_ reduction to formate in a wide potential window. *J. CO2 Util.* **2021**, *51*, 101621.

[17] M. Wang; S. Liu; B. Chen; F. Tian; C. Peng. Synergistic Geometric and Electronic Effects in Bi–Cu Bimetallic Catalysts for CO_2_ Electroreduction to Formate over a Wide Potential Window. *ACS Sustain. Chem. Eng.* **2022**, *10* (17), 5693-5701.

[18] H. Li; X. Yue; Y. Qiu; Z. Xiao; X. Yu; C. Xue; J. Xiang. Selective electroreduction of CO_2_ to formate over the co-electrodeposited Cu/Sn bimetallic catalyst. *Mater. Today Energy* **2021**, *21*, 100797.

[19] H. Ren; X. Wang; X. Zhou; T. Wang; Y. Liu; C. Wang; Q. Guan; W. Li. In-situ constructing Cu_1_Bi_1_ bimetallic catalyst to promote the electroreduction of CO_2_ to formate by synergistic electronic and geometric effects. *J. Energy Chem.* **2023**, *79*, 263-271.

[20] Z. Tao; Z. Wu; X. Yuan; Y. Wu; H. Wang. Copper–Gold Interactions Enhancing Formate Production from Electrochemical CO_2_ Reduction. *ACS Catal.* **2019**, *9* (12), 10894-10898.

[21] Z. Wu; H. Wu; W. Cai; Z. Wen; B. Jia; L. Wang; W. Jin; T. Ma. Engineering Bismuth-Tin Interface in Bimetallic Aerogel with a 3D Porous Structure for Highly Selective Electrocatalytic CO_2_ Reduction to HCOOH. *Angew. Chem.-Int. Ed.* **2021**, *60* (22), 12554-12559.

[22] K. Lakshmanan; W. H. Huang; S. A. Chala; C. Y. Chang; S. T. Saravanan; B. W. Taklu; E. A. Moges; Y. Nikodimos; B. D. Dandena; S. C. Yang; et al. Generating Multi-Carbon Products by Electrochemical CO_2_ Reduction via Catalytically Harmonious Ni/Cu Dual Active Sites. *Small* **2023**, e2307180.

[23] W. Xia; Y. Xie; S. Jia; S. Han; R. Qi; T. Chen; X. Xing; T. Yao; D. Zhou; X. Dong; et al. Adjacent Copper Single Atoms Promote C-C Coupling in Electrochemical CO_2_ Reduction for the Efficient Conversion of Ethanol. *J. Am. Chem. Soc.* **2023**, *145* (31), 17253-17264.

[24] C. Zhu; A. Chen; J. Mao; G. Wu; S. Li; X. Dong; G. Li; Z. Jiang; Y. Song; W. Chen; et al. Cu–Pd Bimetallic Gas Diffusion Electrodes for Electrochemical Reduction of CO_2_ to C_2+_ Products. *Small Struct.* **2023**, *4* (5), 2200328.

[25] L. Han; C.-w. Wang; S.-s. Luo; Y.-t. Zhou; B. Li; M. Liu. Facet effects on bimetallic ZnSn hydroxide microcrystals for selective electrochemical CO_2_ reduction. *Green Energy Environ.* **2024**, *9* (8), 1314-1320.

[26] W. Zhu; L. Zhang; P. Yang; X. Chang; H. Dong; A. Li; C. Hu; Z. Huang; Z. J. Zhao; J. Gong. Morphological and Compositional Design of Pd-Cu Bimetallic Nanocatalysts with Controllable Product Selectivity toward CO_2_ Electroreduction. *Small* **2018**, *14* (7), 1703314.
